# Prevalence of dementia in Latin America and Caribbean countries: Systematic review and meta-analyses exploring age, sex, rurality, and education as possible determinants

**DOI:** 10.1016/j.arr.2022.101703

**Published:** 2022-11

**Authors:** Fabiana Ribeiro, Ana C. Teixeira-Santos, Paulo Caramelli, Anja K. Leist

**Affiliations:** a Department of Social Sciences, University of Luxembourg Belval Campus, 11 Porte des Sciences, L-4366 Esch-sur-Alzette, Luxembourg; b Behavioral and Cognitive Neurology Research Group, Departamento de Clínica Médica, Faculdade de Medicina, Universidade Federal de Minas Gerais, Av. Alfredo Balena, 190 - sala 246, 30130-100 Belo Horizonte, Minas Gerais, Brazil

**Keywords:** Dementia, Prevalence, Meta-analysis, Developing country, Epidemiology, Secular trends

## Abstract

**Background:**

Studies have shown that the prevalence of dementia in Latin America and the Caribbean (LAC) may be higher than in high-income countries. Thus, we sought to systematically analyse the prevalence of dementia and explore possible drivers that lead to this disparity in LAC countries.

**Method:**

We searched Pubmed, Web of Knowledge, Scopus, Lilacs, and SciELO for studies on dementia in LAC countries published in English, Spanish, and Portuguese. Random-effects model was applied.

**Results:**

Thirty-one studies from 15 LAC countries were included. Pooled prevalence of all-cause dementia was 10.66%. Further analyses with studies providing raw prevalence by sex, area, and educational level showed a higher prevalence for women (8.97%) than for men (7.26%). Also, dementia prevalence was higher for rural than urban residents (8.68% vs. 7.71%, respectively). Participants without formal education presented more than double the prevalence of dementia (21.37%) compared to those with at least one year of formal education (9.88%). Studies with more recent data collection showed higher dementia prevalence.

**Conclusion:**

Our findings suggest a high global dementia prevalence in LAC countries and an unequal burden of dementia for women, lower-educated, and rural residents. Secular increases in dementia prevalence call for greater public health efforts for preventative actions.

## Highlights

• Rigorous and most comprehensive review of dementia prevalence in Latin America and the Caribbean (LAC) to date.
• Pooled dementia prevalence estimates are higher in LAC countries compared to high-income countries.
• Higher prevalence of dementia among women and lower-educated adults.
• Higher prevalence among older adults living in rural compared to urban areas.
• Qualitative analyses suggest a modest increase in dementia prevalence in LAC countries over time.

## 1 Introduction

The population living in Latin America and the Caribbean (LAC) countries was estimated to be around 653.962.000 people in 2020 (United Nations Data Retrieval System (n.d.)), accounting for 8.4% of the world population. Population ageing is one of the most crucial global transformations. In 1990, the world's population aged 65 and over was 6%, being 5% in LAC, while by 2019, this number achieved 9% both worldwide and in LAC. Moreover, the proportion of older people is expected to continue increasing, so that one in six people worldwide will be 65 years or older in 2050. Consequently, more cases of dementia are expected to emerge (Custodio et al., 2017).

Dementia defines a range of chronic disorders characterised by cognitive and functional impairment that comprise a variety of specific medical conditions, e.g., vascular dementia, dementia with Lewy bodies, frontotemporal dementia, and Alzheimer's disease; the latter corresponds to 60–70% of cases (World Health Organization, 2020). The Global Burden of Disease Study, using 2016 global data, found that the number of individuals living with Alzheimer's disease and other dementias increased 117% from 1990 to 2016, with a higher prevalence among women than men. Additionally, dementia was the fifth leading cause of death worldwide and consistently ranked among the top three causes of disability in most countries (GBD, Dementia Collaborators, 2016, 2019). In this sense, it is essential to provide robust and accurate figures on the prevalence of dementia since it is indispensable in terms of prognosis for treatment and public policy making. Dementia is one of the most significant causes of dependence among older adults, causing considerable medical and care costs (Custodio et al., 2017, Parra et al., 2018, Prince et al., 2015), which accounts for 0.2% of the total gross domestic product in low- and middle-income countries and 1.4% in rich countries (World Health Organization, 2020). Thus, increases in the prevalence of dementia will boost the difficulties in public health systems of LAC countries, rising the already high economic and social burden related to dementia in this region of the world (Ibáñez et al., 2021), implying significant efforts to adapt social and public policies accordingly (Prince et al., 2013).

Further, dementia prevalence in older age signals the capacity of older adults for financial assistance and support towards younger family members (e.g., through caregiving for grandchildren). In LAC countries, older adults substantially contribute to family income and care when they are healthy, but depend on the support of younger family members when they have a compromised health condition (Gomes, 2007, Pelaez and Martinez, 2002). For this reason, it is essential to evaluate and systematise studies of the prevalence of dementia in LAC countries, taking into account possible differences among countries, which would help them plan for tailored preventive measures and long-term care policies (Custodio et al., 2017, Mukadam et al., 2019).

A previous review performed by Nitrini et al. (2009) assessed the prevalence of dementia in older adults (65–90 + years) in LAC based on eight studies and found no differences in the prevalence compared to developed countries (7.1%). Nevertheless, they pointed out that those participants aged 65–69 years had a higher dementia prevalence, possibly related to the higher number of illiterates among those individuals and to a poor control of cardiovascular risk factors.

A more recent systematic review explored the prevalence of dementia globally between 1980 and 2009, showing that age-standardised prevalence for those aged 60 + years old living in Latin America (11 studies) was higher (8.5%) compared to other world regions, i.e., Europe (6.9%), North America (6.5%), or Asia (from 4.2% to 6.9%). In their review, dementia prevalence increased with age and was higher in women than men, with increasing sex gaps with advancing age (Prince et al., 2013). These sex effects were explained by increased survival in women with dementia, as well as generally increased incidence of dementia among women and men (Brodaty et al., 2012, Carter et al., 2012). Although this study included diverse authors fluent in different languages, their bibliographical search strategy only included terms in English. Furthermore, two recent meta-analyses, one carried out by Cao et al. (2020), including articles in English and Chinese, investigated dementia prevalence in Asia, Africa, Latin America, and Europe/North America. From the 47 selected articles with sex-specific prevalence, only two studies were from Latin America, making valid inferences about dementia prevalence in this world region difficult.

Finally, two systematic reviews specifically exploring the prevalence of dementia in LAC countries revealed discrepant prevalence rates. Zurique Sánchez et al. (2019) included 25 articles published in Spanish or English in their review. They found a general prevalence of 11%, with a higher dementia prevalence in women compared to men and in rural samples compared to urban ones. However, this review did not use strict criteria for the diagnosis of dementia. Instead, the authors included studies in which the dementia diagnosis was based only on screening tests, such as the Mini-Mental State Examination (MMSE). On the other hand, Xiang et al. (2021) used more strict criteria for dementia diagnosis and found a lower prevalence (8%). However, only seven studies, published in English between 2013 and 2018, were included in this review.

As previous systematic reviews on the prevalence of dementia in the older adult population in LAC countries provided limited evidence, this study aimed to deliver a more integrative analysis of the current literature on the prevalence of dementia in LAC countries, strategically searching for articles published in English or in the main official languages of this region, as well as precise estimates of all-cause dementia prevalence, by sex, area (rural and urban), and education, in addition to qualitatively analysing the influence of age, and years of data collection on dementia prevalence.

Regarding the methods carried out in this meta-analysis, we synthesised proportions using the Freeman-Tukey double arcsine transformation, since it is considered an optimal approach. We decided to include this transformation because it has the advantage of stabilising variances and reaching approximate normality over other methods (Freeman and Tukey, 1950, Lin and Xu, 2020). Furthermore, as an advantage, its variances depend only on the sample size, which is fixed and based on known data, while the variances of log and logit transformations also rely on the number of events, which are random variables. In addition, following the Cochrane recommendations (Deeks, Higgins, and Altman, 2022), the robustness of our outcomes was ensured by sensitivity analysis performed by removing studies in which dementia was based on a one-phase diagnostic assessment to explore possible bias from one-versus two-phase assessment, as well as by the leave-one-out method for the pooled prevalence of all-cause dementia, in which one study is removed each time and the analysis repeated to explore the heterogeneity of the included studies and the consistency of results to provide reliable and valid meta-analytical results (Chan and Harky, 2020). Finally, we also provide a descriptive analysis of each study's risk of bias and quality scores. Overall, considering all the used methods, the present systematic review offers a careful and integrated update of the prevalence of dementia in LAC countries.

## 2 Method

We followed the PRISMA guidelines by Moher et al. (2010) and the recommendations of the Cochrane Collaborations for this systematic review and meta-analysis.

### 2.1 Literature search

We carried out a systematic literature search for studies assessing the prevalence of dementia in adults aged 50 or more in LAC countries in five databases: PubMed, Web of Science, Scopus, Lilacs, and SciELO. The first search was carried out on August 16, 2021, and updated on April 13, 2022, in three languages: English, Portuguese, and Spanish, without date restriction. The search terms used and results for each database are provided in the Supplementary Materials. We also examined the reference list of previous systematic reviews assessing the prevalence of dementia in Latin America (Cao et al., 2020, Nitrini et al., 2009, Prince et al., 2013, Xiang et al., 2021, Zurique Sánchez et al., 2019).

### 2.2 Criteria for inclusion and exclusion of studies

The inclusion criteria for articles to be selected in this systematic review were as follows: (i) Observational studies, such as cohort or cross-sectional study designs reporting population- or community-based data from population surveys or patients identified in representative samples of LAC countries; (ii) population-based studies, including the prevalence of dementia in participants aged ≥ 50; and (iii) Studies including frequency of any type of dementia with clearly defined diagnostic criteria, such as with Diagnostic and Statistical Manual of Mental Disorders (DSM), International Statistical Classification of Diseases and Related Health Problems 10th revision (ICD-10), and/or clinical interview performed by trained professionals, and iv) utilization of valid methods to diagnose dementia according to previously cited criteria.

The exclusion criteria of the articles were applied as follows: (i) duplicated studies; (ii) articles including only samples with “mild cognitive impairment”, "cognitive impairment", or “cognitive impairment no dementia”, as they are different syndromes and diagnosed based on different criteria. These studies were excluded because we aimed to select only those studies rigorously defined as dementia with the most consistent samples of participants as possible; (iii) articles focused on sample populations outside LAC; (iv) studies including secondary or tertiary care services in hospitals or clinical-based samples were also excluded to avoid selection bias; and (v) studies not comprising population- or community-based data, such as in-vitro studies, animal studies, case-control studies, randomized control trials, and studies including post-mortem samples; (vi) articles that did not present results of empirical research, such as comments, theoretical, protocol, editorial, book chapters, corrigendum, technical reports, and narrative reviews.

### 2.3 Data extraction and assessment of methodological quality

A standard data form was developed to draw the main estimates from the selected studies. FR extracted data in an Excel spreadsheet for each article, and ACT reviewed the spreadsheet.

The majority of studies only provided diagnostic assessments of all-cause dementia, few studies reported assessment of subtypes of dementia (*n* = 10), and just one specifically assessed Alzheimer's disease. We, thus, only present prevalence estimates of all-type dementia. Figures and estimates collected from each study include the total, number and percentage by sex of respondents with all-type dementia or/and subtypes when provided (e.g., Alzheimer’s disease, vascular dementia, dementia with Lewy bodies); diagnostic criteria and screening instruments for dementia; study design; prevalence rates; essential sociodemographic characteristics when available; such as total sample size, age, sex, and education; and information regarding the rurality of the sample and the socioeconomic status of participants indicated by income.

The methodological quality of the selected studies was assessed by FR and reviewed by ACT through the JBI Critical Appraisal Checklist for Studies Reporting Prevalence Data (Munn et al., 2015), a nine-item checklist designed to evaluate the methodological quality of the studies and to establish possible bias in the design, conduct, and analysis. Any inconsistencies among reviewers about the quality of the articles were resolved by consensus.

### 2.4 Data analysis

In order to estimate the pooled prevalence of all-cause dementia by country, the rate of participants diagnosed with dementia by sample was included in our first analysis. In addition, we also ran study-pooled prevalences of all-cause dementia for sub-groups defined by age range (studies including participants aged ≥ 50 and those including older adults aged ≥ 65), sex, area (urban vs rural), and educational level (no formal education vs at least one year of formal education). In this meta-analysis, participants without formal education and illiterates were categorized into one group called ‘no formal education’.

Our analysis reported the prevalence of dementia (in %) with 95% confidence intervals (CIs). Statistical heterogeneity was evaluated using I^2,^ in which values ≥ 75% were considered an indicator of substantial heterogeneity. Although only studies that used strict diagnostic criteria for dementia assessment were included, high heterogeneity was expected, in line with previous meta-analyses (Bacigalupo et al., 2018, Cao et al., 2020, Zurique Sánchez et al., 2019). Accordingly, the random effects method was used to estimate the pooled prevalence using the Freeman-Tukey double arcsine transformation that stabilizes the variance of each study's proportion (Lin and Xu, 2020).

Sensitivity analyses were performed by removing studies in which dementia was based on a one-phase diagnostic assessment to explore possible bias from one- versus two-phase assessment. The leave-one-out method was also performed to examine the robustness of the findings for the pooled prevalence of all-cause dementia. Finally, we empirically checked publication bias by applying Egger’s regression test and visually creating funnel plots (provided in Supplementary Materials). Moreover, we also calculated, for each study, the women-men risk ratio (RR) to perform a comparative meta-analysis. The RRs were used since they are easier to interpret and less heterogeneous compared to odds ratios. In addition, prevalence estimates were log-transformed before being analysed (Fleiss, 1981, Shadish and Haddock, 1994).

All statistical analyses were carried out in STATA 17, using the METAPROP and METAN commands (StataCorp, 2021).

## 3 Results

### 3.1 Study selection process

**Fig. 1:**
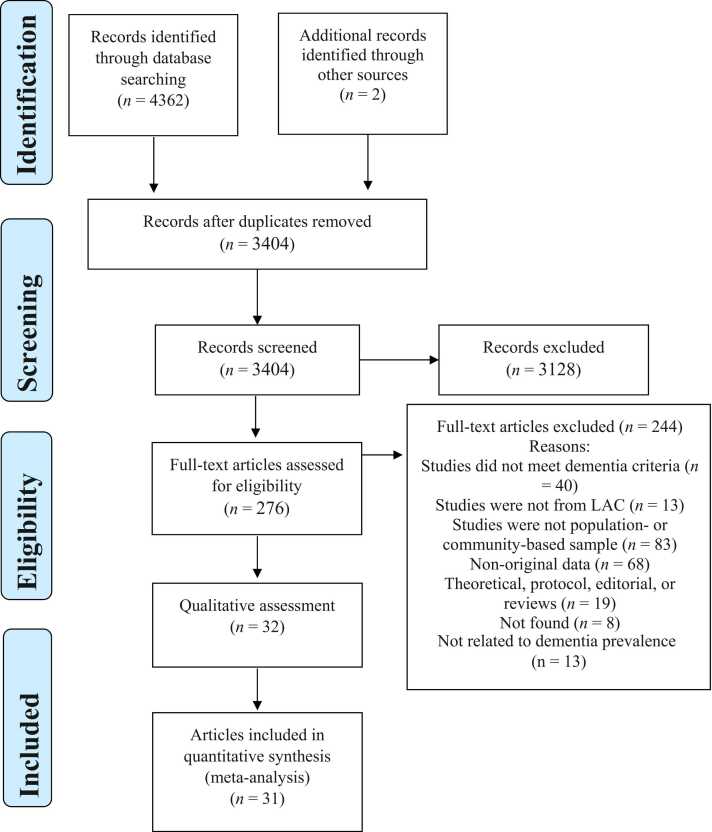
Flow diagram of the study selection process.

The database search yielded 4362 citations, and two additional articles were found in the reference list of Nitrini et al. (2009), resulting in a total of 4364 records. After importing them into Zotero, duplicated studies were filtered. The remaining 3404 citations were independently screened by title and abstract by two independent reviewers (FR and ACT). Following this, 3128 studies were excluded, and 276 articles were selected for full-text screening. There was a good Kappa inter-rater reliability agreement between reviewers over all the records screened *(n* = 3128), including both included and excluded studies (*k* = 0.75) (Fleiss, 1981). The most frequent reasons for exclusion during the full-text screening phase were the study design (not a population- or community-based sampling) (*n* = 83) and non-original data (*n* = 68). A complete list of excluded articles and the reasons for their exclusion are shown in Table S1. Following the full-text, 32 articles were selected and included in the qualitative analysis, while 31 comprised the quantitative analysis. The flow chart of the study selection process and the reason for excluding publication is provided in Fig. 1.

### 3.2 Characteristics of the selected studies

Table 1 summarises the characteristics of the 31 peer-reviewed articles and a conference abstract included in this systematic review. Twenty-five articles and the abstract were published in English, and the remaining six were published in Spanish. In total, they comprise 46 different samples published between 1997 and 2022. Sample sizes ranged from 101 to 18,351 respondents, corresponding to 96,396 participants in total. Sixteen LAC countries were covered: Argentina, Bolivia, Brazil, Chile, Colombia, Cuba, Dominican Republic, Ecuador, Guatemala, Jamaica, Mexico, Panama, Peru, Trinidad and Tobago, Uruguay, and Venezuela, in the qualitative analyses.

**Table 1:** Characteristics of selected studies.

Author and Years of publication (Project)	Data collection year	Country (Region)	Area	Sample Size	Sampling Study Phases	Age range	Educational level	Diagnostic criteria	Diagnostic tools	Screening tools	Prevalence of dementia (n, %)
Albala et al. (1997)^a^	–	Chile (Concepcíon)	Rural/urban	4689	Census, random sample, Two-phases	≥ 65	Urban - Illiterates: n = 814 Literates: 1496 *	DSM-III, NINCS ADRDA	CAMDEX, CDR, laboratory evaluation, and brain computed tomography.	MMSE	174 (3.71%)
Bartoloni et al. (2014) (The Matanza Riachuelo Study)	2012	Argentina (Matanza Riachuelo Basin – Buenos Aires)	Urban	1795	All residents of the region Two-phase study	≥ 60	Education (years): M= 5.6 (SD =3.3)	DSM-IV	MMSE, IADL, ADL, and GDS	–	150 (8.3%)
Bottino et al. (2008)	2002/2003	Brazil (São Paulo)	Urban	1563	Randomly selected from the region Two-phase study	≥ 60	Illiterate (n = 229) 1–4 years (n = 624) 5–8 years (n = 184) 9–11 years (n = 145) ≥ 12 years (n = 291)	DSM-IV	CAMDEX and CAMCOG	MMSE, FOME, IQCODE, B-ADL	107 (6.8%)
Brucki and Nitrini (2014)	2002	Brazil (Mamirauá)	Rural	163	All residents of the region Two-phase study	≥ 50	Illiterates = 110 subjects (67.5%); Group 1 (>0 y) = 53 (32.5%),	DSM-IV	MMSE, BBRC, and VFT	SMI	8 (4.9%)
Caramelli et al. (2011) (The Pietà study)	2007	Brazil (Caeté)	Urban/rural	639	All residents of the region Three-phase study	≥ 75	Illiterates (n = 175, 27.6%) 1–3 years (n = 243, 38.3%) 4–7 years (n = 177,27.9%) 8 + years (n = 40, 6.3%)	DSM-IV	MDRS, AVLT, naming and praxis tests from CERAD, VFT, FAB, PIS, CDR, and CSDD	UPDRS-part III, MMSE, BCSB, PFAQ, FAST, GDS, MINI	175 (27.39%)
César et al. (2016)^b^	2012–2013	Brazil (Tremembe)	Urban/rural	630	Randomly selected from the region One-phase study	≥ 60	Illiterate (n = 87, 13.8%) 1–4 years (n = 348, 55.2%) 5–8 years (n = 83, 13.2%) 9–11 years (n = 51, 8.1%) 12 + (n = 61. 9.7%)	NIA	IQCODE, FAQ, MMSE, delayed recall of the Figure Memory Test of the BCSB, VFT, and CDT	SMI	110 (17.46%)
Correia et al. (2011)	2008/2009	Brazil (Recife)	Urban	101	Unclear Sampling Two-phase study	≥ 65	None (n = 10, 9.2%) 1–4 years (n = 33, 30.3%) 5–8 years (n = 30,27.5%) 9 + (n = 36, 33%)	DSM-IV	Medical examination, CDR, ADL, and MMSE	AD8-Brazil, MMSE, the ADL, and the CDR	15 (14.85%)
Correa-Ribeiro et al. (2013) (FIBRA-RJ Study)	2010/2011	Brazil (Rio de Janeiro)	Urban	683	Randomly selected from private health care plan. Two-phase study	≥ 65	None (n = 25, %) 1–4 years (n = 108, %) 5–8 years (n = 139, %) 9 − 12 (n = 213, %) 13 + (n = 195, %)	DSM-IV, ICD-10, NINCDS-ADRDA, NINDS-AIREN	AVLT, VFT, MDRS, Matrix reasoning, digit span digit symbol-coding, comprehension, and vocabulary (WAIS-III)	MMSE and FAQ	115 (16.9%)
Custodio et al. (2008)	2005	Peru (Lima)	Urban	1532	Randomly selected from the region Three-phase study	≥ 65	Illiterates (n = 269, 17,5%) 1–3 years (n = 312, 20,4%) 4–7 years (n = 417,27,2%) 8 + (n = 534, 34,9%)	DSM IV, NINCDS-ADRDA, NINDS-AIREN	MMSE, CDT, AVLT, Logical Memory Subtest and Letter-Number (WAIS), TMT A and B, ROCF, BNT, WCST, DS, and CDR	MMSE, CDT, PFAQ	103 (6.72%)
Davis et al. (2018)	2011	Trinidad	Urban	1832	Randomly selected from electoral districts One-phase study	≥ 70	None (n = 139, %) Primary (n = 1231, %) Secondary (n = 253, %) Tertiary (n = 124, %)	10/66 dementia diagnosis algorithm/ DSM-IV	CSI-D, CERAD 10-word list learning task, and Euro-D scale	–	442 (23.4%)
Eldemire- Shearer et al. (2018)	2012	Jamaica	Unclear	301	Randomly selected from a national sample. One-phase study	≥ 60	–	DSM-IV	MMSE and medical consensus	–	34 (11.41%)
Gatz et al. (2022)	2017	Bolivia (Tsimane and Moseten villages)	Rural	604		≥ 60	No formal education - Tsimane (80.6%) and Moseten (38.3%)	DSM-V and NIA	VS, DS, W*R*, VFT, SS, and SDT	–	6 (0.99%)
Gooding et al. (2006)	2003–2005	Colombia (Neiva)	Urban	643	Randomly selected from the region Two-phase study	≥ 60	Illiterate (n = 175, 27,2%) 1–3 years (n = 214,33,3%) 4–7 years (n = 169, 26,3%) 8–11 (n = 68, 10,6%) 12 + (n = 17,26%)	GDS, DSM-IV, NINDS-ADRDA and NINDS-AIREN	CERAD, WCST, ROCF, and memory scales (not specified)	MMSE, LIADL, GDS	152 (23.6%)
Herrera et al. (2002)	1996	Brazil (Catanduva)	Urban	1656	Randomly selected from the region Three-phase study	≥ 65	Illiterate (n = 567) 1–3 years (n = 590) 4–7 years (n = 356) 8 + (n = 143)	NINCDS-ADRDA and NINDS-AIREN	MMSE, DS, MF, VFT, abstraction, calculation, construction tests, CDT, LTHT, HIS, and CDR	MMSE, PFAQ	118 (7.13%)
Ketzoian et al. (1997a)^c^	1993–1995	Uruguay (Montevideo)	Urban	2731	All residents of the region Two-phase study	≥ 60	–	DSM	MMSE, NPsyc. evaluation, and medical consensus	Questionnaire	85 (3.1%)
Llibre et al. (2009) (Playa Study on Dementia and Alzheimer’s)	2003	Cuba (Playa)	Urban	18351	Participants registered in all the polyclinics Two-phase study	≥ 65	Illiterate (n = 355, %) ≤ 7 years (n = 8960, %) ≥ 8 years (n = 9036, %)	DSM-IV, NINCDS-ADRDA, NINDS-AIREN	MMSE, CDR, and physical examination	–	1499 (8.2%)
Llibre Rodriguez et al. (2008) (10/66 Study)	2003/2005	Cuba (Havana/Matanzas)	Urban	2937	All residents of the region One-phase study	≥ 65	None (n = 75, 2,5%) Minimal (n = 655, 22,3%) Completed Primary (n = 979, 33,3%) Completed secondary (n = 728, 24,8%) Tertiary (n = 499, 17%)	DSM-IV, 10/66 dementia diagnosis algorithm/ DSM-IV	clinical interview, CSI-D, VFT, and CDR	–	315 (10.72%)
		Dominican Republic (Santo Domingo)	Urban	2009			None (n = 392, 19,7%) Minimal (n = 1022, 51,3%) Completed Primary (n = 370, 18.6%) Completed secondary (n = 135, 6,8%) Tertiary (n = 73, 3,7%)			–	235 (11.7%)
		Peru (Lima/ Canete)	Urban/rural	1932			Urban: None (n = 37, 2,7%) Minimal (n = 90, 6,5%) Completed Primary (n = 460, 33,5%) Completed secondary (n = 481, 35%) Tertiary (n = 305, 22,2%) Rural: None (n = 84, 15,4%) Minimal (n = 141, 25,9%) Completed Primary (n = 267, 49,1%) Completed secondary (n = 36, 6,6%) Tertiary (n = 16, 2,9%)			–	164 (8.49%)
		Venezuela (Caracas)	Urban	1903			None (n = 154, 8,1%) Minimal (n = 438, 23.1%) Completed Primary (n = 950, 50,1%) Completed secondary (n = 263, 13,9%) Tertiary (n = 92, 4,8%)			–	109 (5.73%)
		Mexico (Mexico City/Morelos)	Urban/rural	2002			Urban: None (n = 227, 22,6%) Minimal (n = 354, 35,3%) Completed Primary (n = 229, 22,8%) Completed secondary (n = 99, 9,9%) Tertiary (n = 94, 9,4%) Rural: None (n = 327, 32,7%) Minimal (n = 510, 51%) Completed Primary (n = 122, 12,2%) Completed secondary (n = 25, 2,5%) Tertiary (n = 16,1,6%)			–	170 (8.49%)
Llibre Rodríguez et al. (1999)	1998	Cuba (Marianao)	Urban	779	All Participants registered in polyclinics One phase study	≥ 60	–	DSM-II, NINCDS, ADRDA	MMSE, HIS, and GLDS	–	64 (8.2%)
		Cuba (Bauta)	Urban	340	All Participants registered in polyclinics One phase study	≥ 60	–	DSM-II, NINCDS, ADRDA	MMSE, HIS, and GLDS	–	38 (11.2%)
Lopes et al. (2012)	2000	Brazil (Ribeirao Preto)	Urban	1145	Unclear Sampling Two-phase study	≥ 60	Illiterate (n = 116, 10.1%) 1–4 years (n = 489, 42.7%) 5–8 years (n = 108, 9.4%) ≥ 9 (n = 415, 36.2%)	DSM-IV	CAMDEX and neurological examination	MMSE, FOME, IQCODE, B-ADL	68 (5.94%)
Molero et al. (2007)	1998/2001	Venezuela (Maracaibo, Santa Lúcia) Maracaibo Aging Study	Urban	2438	All residents of the region Three-phase study	≥ 55	M = 5.88 (SD =4.2)	DSM-IV, NINCDS-ADRDA, NINDSAIREN	CDR, DQ, the third part of BDS, LIADL, family history of dementia, and neuropsychiatric evaluation	SPMSQ, LIADL, medical history	196 (8.04% %)
Magalhães et al. (2008)	2005	Brazil (Lagoa Pequena, Santo Estevão)	Rural	466	All residents of the region One-phase study	≥ 60	Literate (n = 118) Illiterate (n = 348)	DSM	CAMDEX, CAMCOG, and neurological evaluation	–	231 (49.57%)
Manrique-Espinoza et al. (2013) (ENSANUT study)	2012	Mexico	Urban/rural	8874	Randomly selected from national sample. One-phase study	≥ 60	None (25.8%) 1–5 years (35.4%) 6 year of education (18.3%) 7–9 years of education (9.0%) ≥ 10 years (11.5%)	DSM-IV	VFFT, Mini-cog, CES-D, ADL, and LIADL	–	612 (7.9%)
Martínez Querol et al. (2005)	2003	Cuba (Playa)	Urban	2139	Participants registered in the polyclinic One-phase study	≥ 65	–	DSM-IV and NINCDS-ADRDA	MMSE, CDR, HIS, and Medical consensus	–	116 (5.42%)
Mejia-Arango et al. 2011 (The Mexican Health and Aging Study)	2001	Mexico (Mexico City)	Urban/rural	7166	All residents of the region One-phase study	≥ 60	None (n = 2138, 36%) 1–6 years (n = 3646, 50.3%) ≥ 7 years (n = 1038, 13.7%)*	DSM IV	CCCE, IQCODE, IADLs, and BADL	–	357 (4.98%)
Mejia-Arango et al. (2021) (The Mexican Health and Aging Study)	2015	Mexico (Mexico City)	Urban/rural	10219	All residents of the region One-phase study	≥ 60	None (n = 2142, 24%) 1–6 years (n = 5541, 52.9%) ≥ 7 years (n = 2536, 22.9%)	NINCDS-ADRDA	CCCE, IQCODE, IADLs, and BADL	–	751 (7.30%)
Neita et al. (2014)	2010	Jamaica (Kingston)	Urban	200	Randomly selected from the region Two-phase study	≥ 60	–	DSM-IV	Clinical and Diagnostic Assessment Procedure	MMSE	13 (6.5%)
Pedraza et al. (2017)	2012/2014	Colombia (Bogotá)	Urban	1235	Unclear Sampling Two-phase study	≥ 50	Primary (n = 260, 61.4%) Incomplete high school (n = 64, 15.1%) Complete high school (n = 31, 15.8%) Higher education (n = 67, 15.8%)	DSM-IV	MoCA, MMSE, HIS, CDT, GMT, VFT, the 64-item naming test, ROCF, TMT-A, KFBI, and LIADL	SMI, BI, SMCQ, ZBI, MoCA Test, MMSE, GDS	280 (23%)
Ramos-Cerqueira et al. (2005)	2000	Brazil (Piraju)	Urban	2222	Unclear Sampling. Two-phase study	≥ 65	–	DSM-IV and CDR	Presence of symptoms such as forgetfulness and subsequent learning and language problems and thought and orientation disturbances, and medical consensus	–	45 (2.02%)
Sanchez-Arenas et al. (2013) (Study on aging and dementia in Mexico-SADEM)	2009/2010	Mexico (Mexico City)	Urban	2905	Randomly selected from participants registered in Social Security One-phase study	≥ 60	None (n = 368) 1–6 years (n = 1575) ≥ 7 years (n = 962)	DMS-IV and NINCDS-ADRDA	ADAS, RBMT, SKT, BDS, VFT, CDT, CERAD, CDR, CES-D, and consensus by geriatricians	EuroQoL-5, ADL, IADLs, MMSE, SF-36, MIS	109 (3.4%)
Scazufca et al. (2008) (The Sao Paulo Ageing & Health Study)	2003/2005	Brazil (Butantã – São Paulo)	Urban	2068	All residents Living in a disadvantaged region One-phase study	≥ 65	None (n = 793, 38.3%) 1–3 years (n = 1083, 52.3%) ≥ 4 years (n = 196, 9.4%)	DSM-IV, HAS-DDS	CSI-D, CERAD, and HAS-DDS	–	105 (5.07%)
Soto-Añari et al. (2021)	2020	Argentina (city unspecified)	Unclear	511	non-probabilistic sample from private and public clinics. Two-phase	≥ 60	Illiterate/primary school (n = 40, 7.8%) High school (n = 255, 49.9%) Technical formation (76, 14.9%) College (n = 110, 21.5%) Master/PhD (n = 30, 5.9%)	NIA	Consensus by neuropsychologists, and neurologists	T-MoCA AD8, GDS	40 (7.83)
		Bolivia		565			Illiterate/primary school (n = 146 (25.8) High school (n = 229, 40.5%) Technical formation (46, 8.1%) College (n = 113, 20.0%) Master/PhD (n = 31, 5.5%)				161 (28.50)
		Chile		472			Illiterate/primary school (n = 54, 11.4%) High school (n = 225, 47.7%) Technical formation (n = 65, 13.8%) College (n = 104, 22.0%) Master/PhD (n = 24, 5.1%)				76 (16.10)
		Colombia		712			Illiterate/primary school (n = 125, 17.7) High school (n = 302, 42.4%) Technical formation (65, 9.1%) College (n = 168, 23.6%) Master/PhD (n = 52, 7.3%)				92 (12.92)
		Ecuador		436			Illiterate/primary school (n = 77, 17.7%) High school (n = 177, 40.6%) Technical formation (n = 33, 7.6%) College (n = 119, 27.3%) Master/PhD (n = 30, 6.9%)				72 (16.51)
		Guatemala		340			Illiterate/primary school (n = 49, 14.4%) High school (n = 127, 37.4%) Technical formation (n = 32, 9.4%) College (n = 106, 31.2%) Master/PhD (n = 26, 7.6%)				58 (17.06)
		Mexico		648			Illiterate/primary school (n = 93, 10.5%) High school (n = 215, 33.2%) Technical formation (n = 139, 21.5%) College (n = 134, 20.7%) Master/PhD (n = 67, 10.3%)				67 (10.34)
		Peru		601			Illiterate/primary school (n = 186, 30.9%) High school (n = 235, 38.1%) Technical formation (n = 36, 6.0%) College (n = 120, 20.0%) Master/PhD (n = 24, 4.0%)				144 (23.96)
		Dominican Republic		384			Illiterate/primary school (n = 88, 22.9%) High school (n = 114, 29.7%) Technical formation (35, 9.1%) College (n = 112, 29.2%) Master/PhD (n = 35, 9.1%)				45 (11.72)
		Venezuela		576			Illiterate/primary school (n = 22, 3.8%) High school (n = 222, 38.5%) Technical formation (n = 59, 10.2%) College (n = 177, 30.7%) Master/PhD (n = 96, 16.7%)				63 (10.94)
Villarreal et al. (2016) Panama Aging Research Initiative study	2012/2013	Panama (Panama City)	Unclear	259	Participation was offered to every adult who attended the geriatric service of a public hospital One-phase study	≥ 65	Alzheimer’s disease M = 6.9 (SD= 3.6) years Control M = 8.5 (SD= 4.0) years	NINCDS-ADRDA criteria	Consensus by geriatricians	B-ADL, IADLs, MMSE, CDT, GDS (GLDS)	31 (11.97%)

Note: AD8: Brazil: Brazilian versions of the Clinical Dementia Rating, ADAS: Alzheimer’s Disease Assessment Scale, ADL: Katz Index of Independence in Activities of Daily Living, ADRDA: Alzheimer Disease and Related Disorders, AVLT: Rey Auditory Verbal Learning Test, B-ADL: Bayer-Activities of Daily Living Scale, BADLs: Basic activities of daily living, BCSB: Brief Cognitive Screening Battery, BDS: Blessed Dementia Scale, BI: The Barthel Index, BNT: Boston Naming Test, CAMCOG: Cambridge Cognitive Examination, CAMDEX: Cambridge Examination for Mental Disorders, CCCE: Cross Cultural Cognitive Examination, CDR: Clinical Dementia Rating, CDT: clock drawing test, CERAD: Consortium To Establish A Registry For Alzheimer’s Disease, CES-D: Epidemiologic Studies Depression Scale, CFT: consensus on clinical diagnostic criteria of Frontotemporal lobar, CSDD: Cornell scale for depression in dementia, CSI-D: Community Screening Instrument for Dementia, DLB: Consensus guidelines for the clinical pathologic diagnosis of dementia with Lewy bodies, DQ: Dementia Questionnaire, DS: Digit Span, DSM: Diagnostic and Statistical Manual of Mental Disorders, FAB: Frontal Assessment Battery, FAQ: Functional Activities Questionnaire, FAST: Functional Assessment Staging, FOME: Fuld Object Memory Evaluation, GDS: Geriatric Depression Scale, GLDS: Global Deterioration Scale, GMS: Geriatric Mental State, GMT: Grober-Buschke memory test, HAS-DDS: History and Aetiology Schedule Dementia Diagnosis and Subtype, HIS: Hachinski Ischemic Scale, IADLs: instrumental activities of daily living, IQCODE: Questionnaire on Cognitive Decline in the Elderly, KFBI: Kertesz Frontal Behavioral Inventory, LIADL: Lawton and Brody Instrumental Scale, LTHT: Luria three hand position test, MDRS: Mattis Dementia Rating Scale, MF: Memory of figures, MINI: Mini International Neuropsychiatric Interview, MIS: Memory Impairment Screen, MMSE: Mini Mental State Examination, MNA: Mini-Nutritional Assessment, MOCA: Montreal Cognitive Assessment, NIA: National Institute on Aging, NINCDS-ADRDA: National Institute of Neurological Communicative Disorders and Stroke- Alzheimer’s Disease and Related Disorders Association, NINDS-AIREN: The National Institute of Neurological Disorders and Stroke– Association Internationale pour la Recherche et l’Ensignement en Neurosciences, PFAQ: Pfeffer Functional Activities Questionnaire, PIS: Physical and Instrumental-Self Maintenance scale, RBMT: The Rivermead Behavioural Memory, ROCF: Rey–Osterrieth complex figure, DST: Stick design test, SKT: The Syndrom Kurztest, SF-36: Short Form of the quality of life survey, SMCQ: subjective memory complaints questionnaire, SMI: Subjective memory impairment, SPMSQ: Short Portable Mental Status Questionnaire, SS: Spatial Span, T-MoCA: Telephone version of Montreal Cognitive Assessment, TMT: Trail Making Test, UPDRS-part III: Unified Parkinson’s Disease Rating Scale, VFT: Verbal fluency tests, VS: Visual Scan, WAIS: Wechsler Adult Intelligence Scale, WCST: The Wisconsin Card Sorting Test, W*R*: Word Recall, ZBI: Zarit Burden Interview

Note: a - Data sent by the study author; b – Some information was retrieved from Cesar (2014) Doctoral thesis; c - Additional information retrieved from Nitrini et al. (2009) and Ketzoian et al. (1997b) Poster.

Regarding rurality, most of the studies included participants in urban areas (*n* = 23), while two studies from Brazil (Brucki and Nitrini, 2014, Magalhães et al., 2008) and one from Bolivia (Gatz et al., 2022) comprised individuals from rural populations, and eight studies had individuals recruited in both, rural and urban areas. In one article (Soto-Añari et al., 2021), the rurality of the samples was unclear for the samples from all nine countries.

A total of ten articles specified the types of dementia, such as Alzheimer’s disease and vascular dementia (Bottino et al., 2008, Correa Ribeiro et al., 2013, Custodio et al., 2008; Gatz et al., 2022; Herrera et al., 2002; Llibre Rodríguez et al., 1999; Maestre et al., 2018; Martínez Querol et al., 2005; Scazufca et al., 2008; Villarreal et al., 2016).

Studies differed on whether they assessed dementia in one phase, with a one-time full assessment or two or more phases, with an initial screening phase and subsequent in-depth assessment of participants with probable dementia. Fourteen out of the 32 included studies conducted a two-phase diagnosis (Albala et al., 1997, Bartoloni et al., 2014, Bottino et al., 2008, Brucki and Nitrini, 2014, Correa Ribeiro et al., 2013, Correia et al., 2011, Gooding et al., 2006, Ketzoian et al., 1997a, Llibre et al., 2009, Lopes et al., 2012, Neita et al., 2014, Pedraza et al., 2017, Ramos-Cerqueira et al., 2005, Soto-Añari et al., 2021) and four studies conducted more than two phases-diagnosis to identify patients with dementia (Caramelli et al., 2011, Custodio et al., 2008, Herrera et al., 2002, Molero et al., 2007). In general, MMSE was used as the main instrument to screen symptoms of dementia; however, the following phases varied widely among studies in terms of instruments used.

As provided in Table 1, the Diagnostic and Statistical Manual of Mental Disorders (DSM-III, DSM-IV, or DSM-V) was the most used criteria for diagnosing dementia. However, some studies used the 10/66 algorithm in addition to the DSM criteria (Davis et al., 2018; Llibre Rodriguez et al., 2008). Besides the DSM, one study used the History and Aetiology Schedule Dementia Diagnosis and Subtype (Scazufca et al., 2008), another two applied the National Institute on Aging criteria (César et al., 2016; Gatz et al., 2022; Soto-Añari et al., 2021), and one of them used the cut-off points of the Cambridge Examination for Mental Disorders (Magalhães et al., 2008).

Some studies aimed specifically to diagnose Alzheimer’s disease, and in this case, the search revealed nine studies using the criteria established by the National Institute of Neurological and Communication Disorders and Stroke and the Alzheimer’s Disease and Related Disorders Association (NINCDS-ADRDA) (Albala et al., 1997, Correa Ribeiro et al., 2013, Custodio et al., 2008, Gooding et al., 2006, Herrera et al., 2002, Llibre et al., 2009, Llibre Rodríguez et al., 1999, Molero et al., 2007, Martínez Querol et al. 2005, Sanchez-Arenas et al., 2013, Villarreal et al., 2016). In the case of vascular dementia, the diagnosis was established based on criteria from the National Institute of Neurological Disorders and Stroke Association Internationale pour la Recherche et l’Enseignement en Neurosciences (NINDS-AIREN) (Correa Ribeiro et al., 2013; Custodio et al., 2008, Gooding et al., 2006, Herrera et al., 2002, Llibre et al., 2009, Molero et al., 2007). As Villarreal et al. (2016) assessed only Alzheimer’s Disease, we excluded it from the quantitative analyses of all-cause dementia.

### 3.3 Methodological quality of the included studies

The methodological rigour of each study was assessed by applying the nine criteria provided by the JBI critical appraisal checklist for studies reporting prevalence data. As shown in Table 2, only studies meeting a minimum of five criteria were included in the quantitative analysis. Furthermore, one of the criteria was related to the dementia diagnosis assessment, which should consist of a valid method for identifying dementia. This was a mandatory criterion in itself for a study to be included in this systematic review. Specifically, nine articles had total scores on the JBI critical appraisal checklist (Bottino et al., 2008, César et al., 2016, Custodio et al., 2008, Davis et al., 2018, Llibre et al., 2009, Llibre Rodriguez et al., 2008, Molero et al., 2007, Scazufca et al., 2008),

**Table 2:** JBI critical appraisal checklist assessment results.

Study Author and Year	Questions									
	Was the sample representative?	Were participants appropriately recruited?	Was the sample size adequate?	Are the subjects and setting described in detail?	Data analysis conducted with sufficient sample coverage?	Have valid methods been used for dementia identification?	Was dementia measured in a standard way?	Was there appropriate statistical analysis?	Was the response rate adequate?	Score
Albala et al. (1997)^a^	Yes	Yes	Yes	No	No	Yes	Unclear	No	Yes	5
Bartoloni et al. (2014)	No	Yes	Yes	Yes	Yes	Yes	Yes	No	Yes	7
Bottino et al. (2008)	Yes	Yes	Yes	Yes	Yes	Yes	Yes	Yes	Yes	9
Brucki and Nitrini (2014)	Yes	Unclear	Yes	Yes	Yes	Yes	Yes	No	Unclear	5
Caramelli et al. (2011)	Yes	Yes	Yes	Yes	Yes	Yes	Yes	No	Unclear	7
César et al. (2016)^b^	Yes	Yes	Yes	Yes	Yes	Yes	Yes	Yes	Yes	9
Correa Ribeiro et al. (2013)	No	Yes	Yes	Yes	Yes	Yes	Yes	Yes	Yes	7
Correia et al. (2011)	Yes	Unclear	Yes	Yes	Yes	Yes	Yes	Yes	Yes	8
Custodio et al. (2008)	Yes	Yes	Yes	Yes	Yes	Yes	Yes	Yes	Yes	9
Davis et al. (2018)	Yes	Yes	Yes	Yes	Yes	Yes	Yes	Yes	Yes	9
Eldemire-Shearer et al. 2018	Yes	Yes	Yes	No	Yes	Yes	Unclear	No	Yes	6
Gatz et al. (2022)	Yes	Yes	Yes	Yes	Yes	Yes	Yes	Yes	Yes	9
Gooding et al. (2006)	Yes	Yes	Yes	Yes	Yes	Yes	Yes	No	Unclear	7
Herrera et al. (2002)	Yes	Yes	Yes	Yes	Yes	Yes	Yes	No	Yes	8
Ketzoian et al. 1997a^c^	Yes	Yes	Yes	No	Yes	Yes	Yes	No	Unclear	6
Llibre et al. (2009)	Yes	Yes	Yes	Yes	Yes	Yes	Yes	Yes	Yes	9
Llibre Rodríguez et al., 1999	Yes	Yes	Yes	No	Yes	Yes	Yes	Yes	Unclear	7
Lopes et al. (2012)	Yes	Unclear	Yes	Yes	Yes	Yes	Yes	Yes	Yes	8
Llibre Rodriguez et al., 2008	Yes	Yes	Yes	Yes	Yes	Yes	Yes	Yes	Yes	9
Molero et al. (2007)	Yes	Yes	Yes	Yes	Yes	Yes	Yes	Yes	Yes	9
Magalhães et al. (2008)	Yes	Yes	Yes	Yes	Yes	Yes	Unclear	Yes	Yes	8
Manrique-Espinoza et al. (2013)	Yes	Yes	Yes	Yes	Yes	Yes	Yes	No	Unclear	7
Martínez Querol et al. (2005)	Yes	Yes	Yes	Yes	Yes	Yes	Unclear	No	Unclear	6
Mejia-Arango and Gutierrez (2011)	Yes	Yes	Yes	Yes	Yes	Yes	Unclear	Yes	Yes	8
Mejia-Arango et al. (2021)	Yes	Yes	Yes	Yes	Yes	Yes	Unclear	Yes	Yes	8
Neita et al. (2014)	Yes	Yes	Yes	No	Yes	Yes	Unclear	No	Unclear	5
Pedraza et al. (2017)	Unclear	Unclear	Yes	Yes	Yes	Yes	Unclear	Yes	Unclear	5
Ramos-Cerqueira et al. (2005)	Yes	Unclear	Yes	No	No	Yes	Yes	No	Yes	5
Sanchez-Arenas et al., 2013	Yes	Yes	Yes	Yes	Yes	Yes	Unclear	Yes	Unclear	7
Scazufca et al. (2008)	Yes	Yes	Yes	Yes	Yes	Yes	Yes	Yes	Yes	9
Soto-Añari et al. (2021)	No	No	Yes	Yes	Yes	Yes	Yes	Yes	Yes	7
Villarreal et al. (2016)	No	Yes	Yes	Yes	Yes	Yes	Unclear	No	Unclear	5

Note: a - Data sent by the study author; b – Some information was retrieved from Cesar (2014) Doctoral thesis; c - Additional information retrieved from Nitrini et al. (2009) and Ketzoian et al. (1997b) Poster.

All but five studies (Bartoloni et al., 2014, Correa Ribeiro et al., 2013, Pedraza et al., 2017, Soto-Añari et al., 2021, Villarreal et al., 2016) did not include representative samples to address the target population. Bartoloni et al. (2014) excluded illiterate participants from the final sample, limiting the sample’s representativeness, since the illiteracy rate is high in older adults in LAC countries (UNESCO, 2017). Correa Ribeiro et al. (2013) only included participants from a private health care plan, representing less than 30% of the older adults in Rio de Janeiro at the time of data collection (Agência Nacional de Saúde, 2021). The description of the study methods by Pedraza et al. (2017) did not conclusively state whether the sample was representative, and Villarreal et al. (2016) comprised older adults of just one public hospital in Panama, for this reason, their results may underestimate the extent of dementia among older Panamanians (Villarreal et al., 2019). Although five articles were classified as unclear regarding the appropriateness of recruited participants (Brucki and Nitrini, 2014, Correia et al., 2011, Lopes et al., 2012, Pedraza et al., 2017, Ramos-Cerqueira et al., 2005), all articles presented adequate sample size. Further, in 10 studies, it was not clear whether dementia was assessed in a standardised way, i.e., whether those involved in data collection were trained in the use of the instruments (Albala et al., 1997; Eldemire-Shearer et al., 2018; Magalhães et al., 2008; Martínez Querol et al., 2005; Mejia-Arango and Gutierrez, 2011; Mejia-Arango et al., 2021; Neita et al., 2014; Pedraza et al., 2017; Sanchez-Arenas et al., 2013; Villarreal et al., 2016). Finally, Soto-Añari et al. (2021) included a non-probabilistic sampling obtained by convenience samples from private and public clinics. Furthermore, they excluded participants with a positive diagnosis of COVID-19, depression, or anxiety during the period of social restrictions.

Regarding the appropriateness of the statistical analysis, 13 articles did not report confidence intervals (Albala et al., 1997, Bartoloni et al., 2014, Brucki and Nitrini, 2014, Caramelli et al., 2011; Eldemire-Shearer et al., 2018; Gooding et al., 2006; Herrera et al., 2002; Ketzoian et al., 1997a; Manrique-Espinoza et al., 2013; Martínez Querol et al., 2005; Neita et al., 2014; Ramos-Cerqueira et al., 2005; Villarreal et al., 2016).

### 3.4 Quantitative analyses

**Fig. 2:**
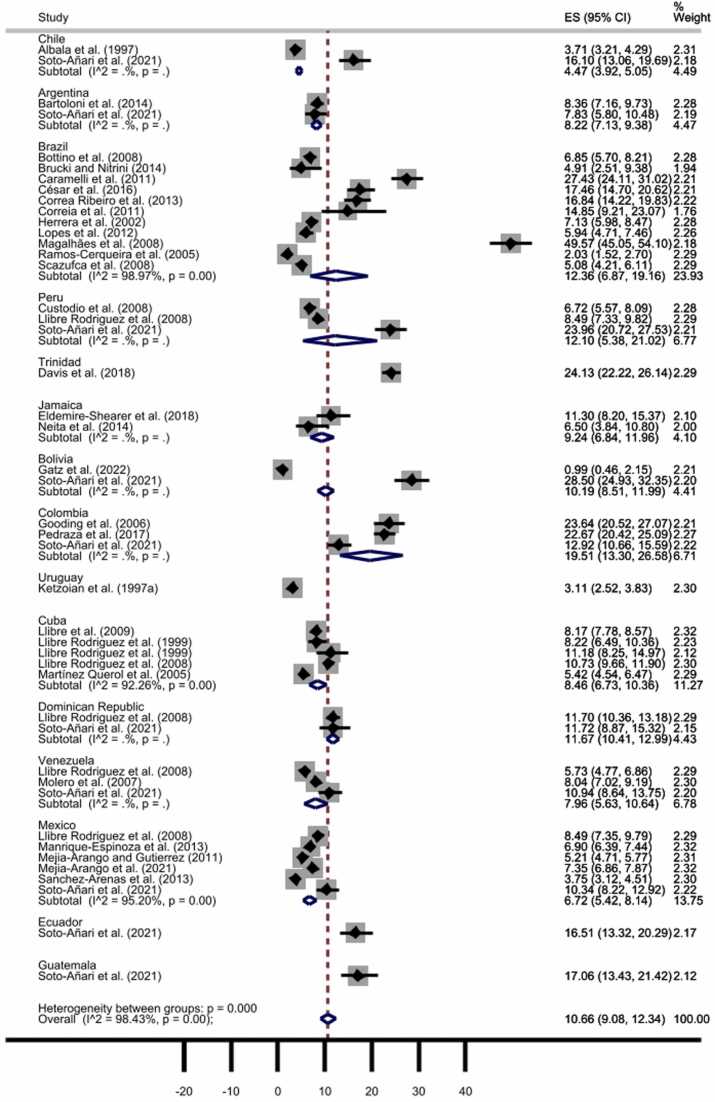
All-cause dementia prevalence by countries. The diamonds represent the prevalence of all-cause dementia, the horizontal bars the 95% confidence intervals. Note: ES: effect size; CI: confidence intervals.

As displayed in Fig. 2, the pooled prevalence of all-cause dementia throughout the 31 included studies, comprising a total of 45 samples, was 10.66% (95% CI: 9.08–12.34%). However, we detected statistically significant heterogeneity between studies (I^2^ = 98.43%, *p* < 0001) and significant publication bias by Egger’s test (t = 2.58, *p* = 0.009) (the funnel plot is available as Figure S1A), which pointed to the need for further analyses to explore the source of heterogeneity.

In a second step, the prevalence of all-cause dementia was pooled after excluding the studies with one-phase diagnosis. A prevalence of 10.76% (95% CI: 8.54–13.21%) was observed (Fig. S2), with no publication bias (*t* = 1.74, *p* = 0.08) (for the funnel plot, see Fig. S1B).

**Fig. 3:**
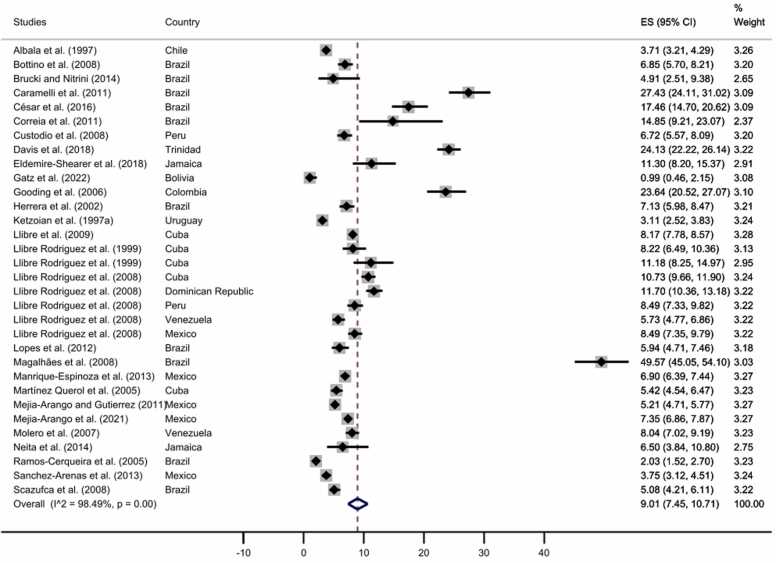
All-cause dementia prevalence for studies with representative samples. The diamonds represent the prevalence of all-cause dementia for studies with representative samples, the horizontal bars the 95% confidence intervals. Note: ES: effect size; CI: confidence intervals.

Moreover, we performed an analysis including only studies with representative samples; for this, four studies were excluded (Bartoloni et al., 2014, Correa Ribeiro et al., 2013, Pedraza et al., 2017, Soto-Añari et al., 2021), and the prevalence was 9.01% (95% CI: 7.45–10.71%), (Fig. 3, for funnel plot, see Fig. S1C).

**Fig. 4:**
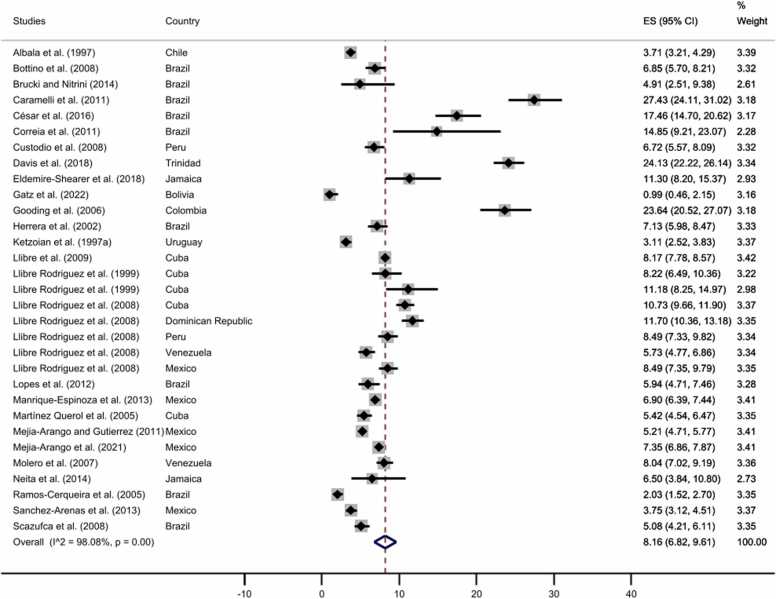
All-cause dementia prevalence after performing the leave-one-out method. The diamonds represent the prevalence of all-cause dementia for studies with representative samples, the horizontal bars the 95% confidence intervals. Note: ES: effect size; CI: confidence intervals.

We also used the leave-one-out method to observe if any of the studies was influencing the prevalence. Here, only Magalhães et al. (2008) study increased the pooled prevalence of all-cause dementia; the difference between this study and the remaining ones is that the former only included participants from rural areas. After excluding this article, the pooled prevalence decreased to 8.16%, with no publication bias (*t* = 1.60, *p* = 0.10) (see Fig. 4; for the funnel plot, see Fig. S1D). Furthermore, as expected, the pooled estimate of all-cause dementia prevalence in representative studies including participants aged 65 years or more (8.96%, 95% CI: 6.67–11.55%) was higher than those comprising older adults aged 50 years or more (7.43%, 95% CI: 5.92–9.09%) (see Fig. S3). No publication bias was observed for any of the two analyses, *t* = 1.47, *p* = 0.14; *t* = 1.43, *p* = 0.15, respectively.

**Fig. 5:**
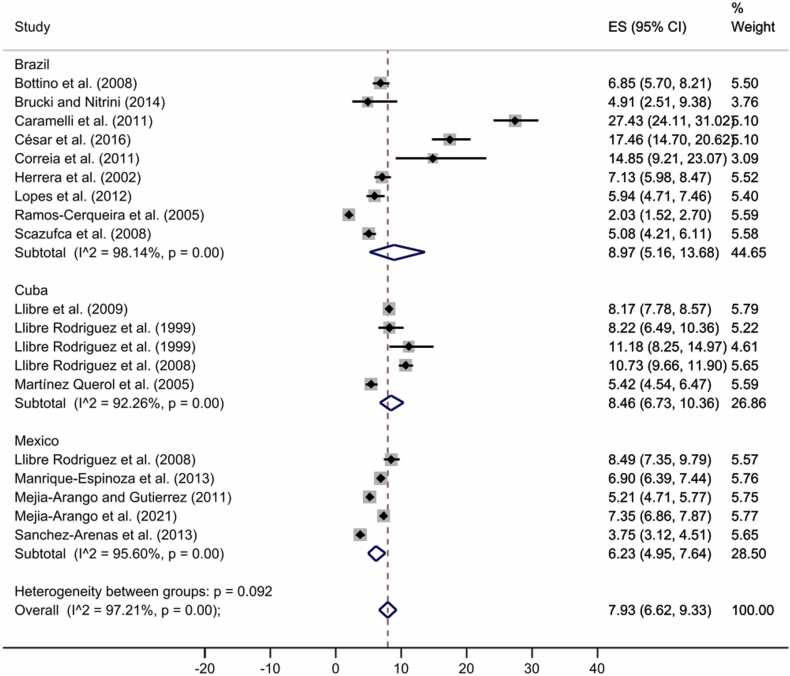
All-cause dementia prevalence by countries in representative samples. The diamonds represent the prevalence of all-cause dementia for studies with representative samples, the horizontal bars the 95% confidence intervals. Note: ES: effect size; CI: confidence intervals.

In addition, we also pooled the prevalence for the following countries, Mexico, Brazil, and Cuba, since each of them had prevalence estimates from more than three articles. Their pooled prevalence was 7.93% (95% CI: 6.62–9.33%) (Fig. 5, funnel plot Fig. S1E), with no publication bias (*t* = 1.94, *p* = 0.05).

In the next step, we performed four sub-group meta-analyses, with only representative studies, stratifying by sex and including only those studies with enough information (n = 23 for the analyses for each sex, specifically for same-sex participants). We excluded Magalhães et al. (2008) as the leave-one-out from the previous section pointed out that this study considerably increased the overall estimate.

**Fig. 6:**
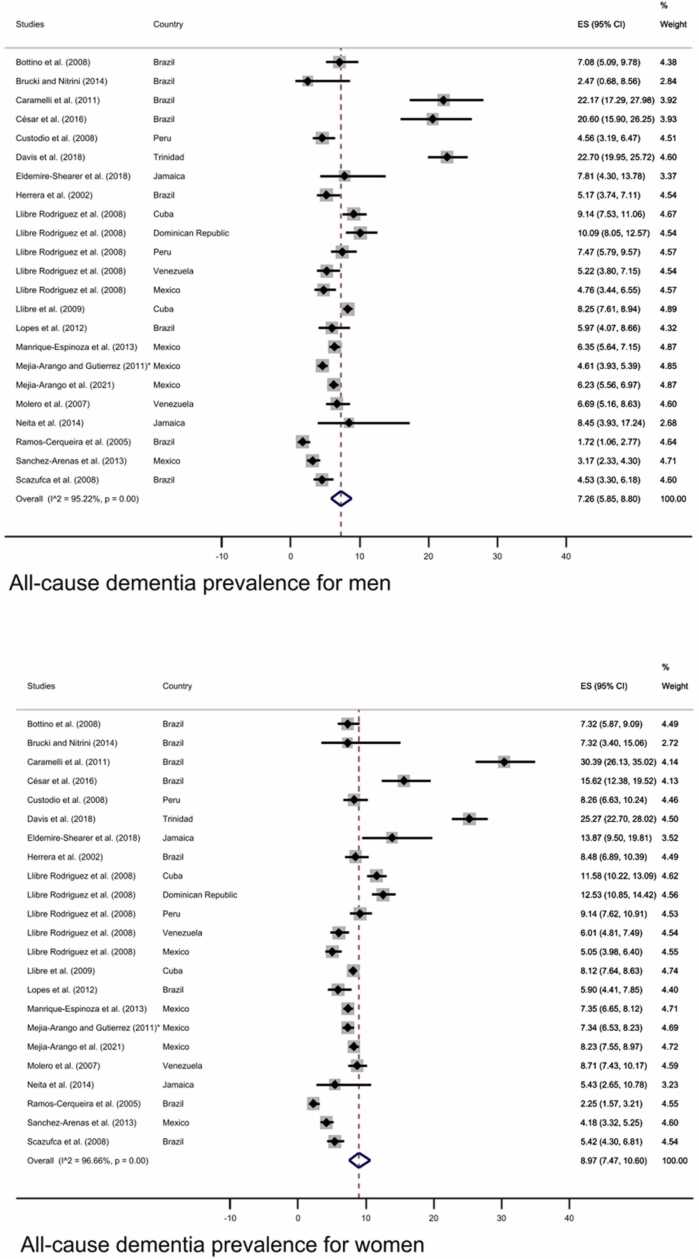
All-cause dementia prevalence in men (upper panel) and women (lower panel). The diamonds represent the prevalence of all-cause dementia for studies with representative samples, the horizontal bars the 95% confidence intervals. Note: ES: effect size; CI: confidence intervals; * Additional information retrieved from Mejia-Arango et al. (2021).

As displayed in Fig. 6, the sub-group analyses exploring prevalence of dementia by sex showed a higher prevalence for women (8.97%, 95% CI: 7.47–10.60%) than men (7.26%, 95% CI: 5.84–8.80%) (funnel plot Fig. S4A-B).

**Fig. 7:**
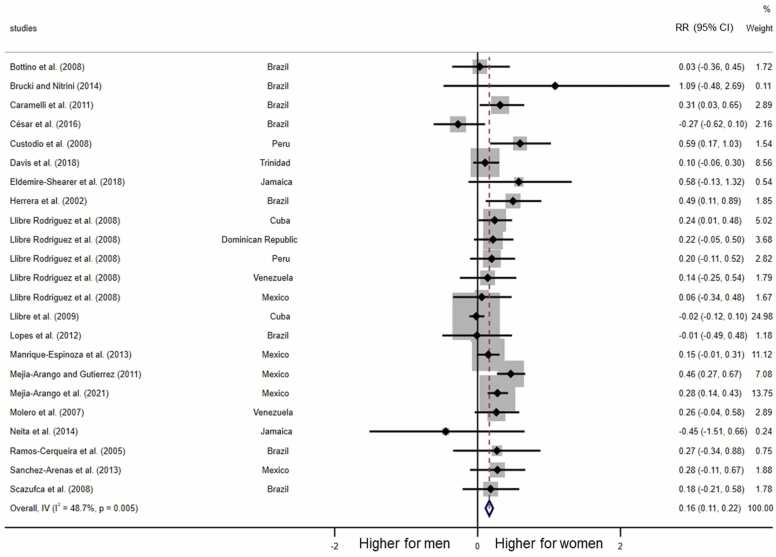
Meta-analysis of female disadvantage in all-type dementia prevalence. Note: RR: Log-transformed Risk Ratios.

As shown in Fig. 7, all but two studies showed female disadvantage in the relative risk ratio of dementia prevalence. In total, there was a statistically significant higher prevalence of dementia among women (RR = 0.16, 95% CI = 0.11–0.22, *p* < 0.001).

**Fig. 8:**
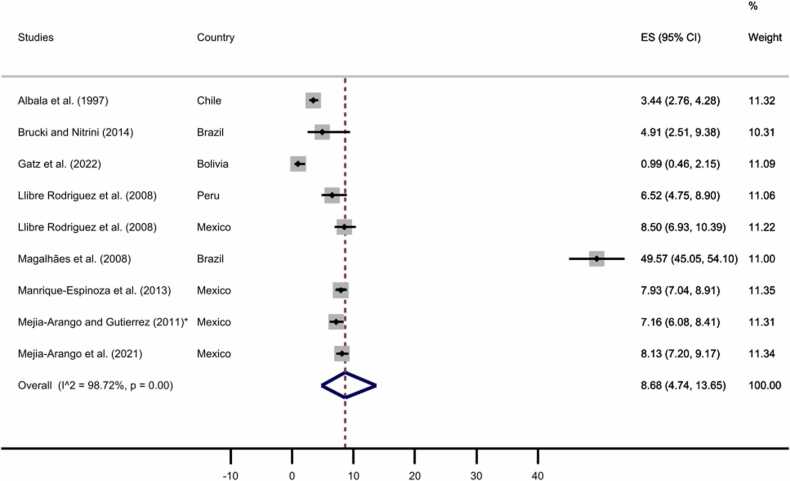
All-cause dementia prevalence by rural area. The diamonds represent the prevalence of all-cause dementia for studies with representative samples, the horizontal bars the 95% confidence intervals. Note: ES: effect size; CI: confidence intervals.

**Fig. 9:**
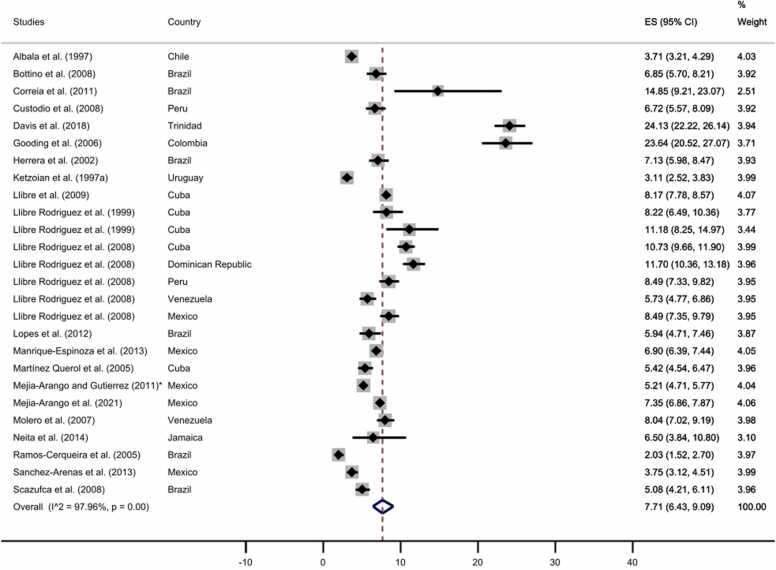
All-cause dementia prevalence by urban area. The diamonds represent the prevalence of all-cause dementia for studies with representative samples, the horizontal bars the 95% confidence intervals. Note: ES: effect size; CI: confidence intervals; * Additional information retrieved from Mejia-Arango et al. (2021).

Furthermore, we carried out two separated subgroup meta-analyses for rural and urban samples. Nine studies were based on rural samples, while 26 studies were from urban samples. One of the articles previously excluded due to a fully rural sample (Magalhães et al., 2008), was included in the analysis to estimate the pooled prevalence in the rural area. As displayed in Fig. 8, Fig. 9, the pooled prevalence of all-cause dementia was 8.68% for rural areas (95% CI: 4.74–13.65%) and 7.71% for urban areas (95% CI: 6.43–9.09%; see Fig. S4C-D for funnel plots).

* Additional information retrieved from Mejia-Arango et al. (2021).

**Fig. 10:**
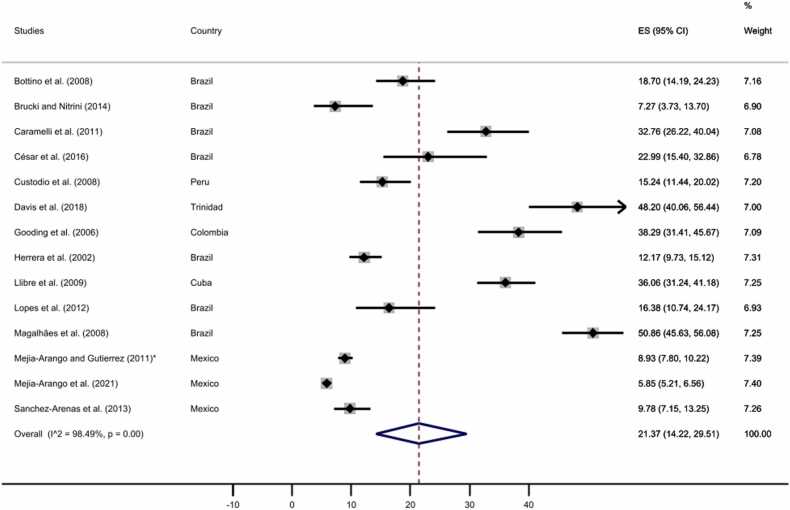
All-cause dementia prevalence for participants with no formal education. The diamonds represent the prevalence of all-cause dementia for studies with representative samples, the horizontal bars the 95% confidence intervals. Note: ES: effect size; CI: confidence intervals; * Additional information retrieved from Mejia-Arango et al. (2021).

**Fig. 11:**
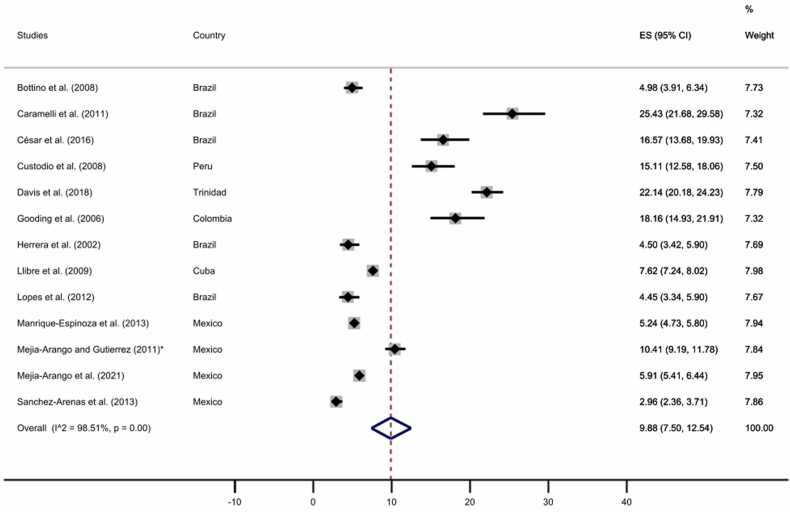
All-cause dementia prevalence for participants with at least one year of formal education. The diamonds represent the prevalence of all-cause dementia for studies with representative samples, the horizontal bars the 95% confidence intervals. Note: ES: effect size; CI: confidence intervals; * Additional information retrieved from Mejia-Arango et al. (2021).

Finally, we performed two separated subgroups meta-analyses comprising those studies with complete data for the educational level to explore the dementia prevalence for no formal education and at least one year of formal education. As displayed in Fig. 10, Fig. 11, our analyses revealed a prevalence of 21.37% (95% CI: 14.22–29.51%) for those participants without formal education and a prevalence of 9.88% (95% CI: 7.50–12.54%) for those with at least one year of formal education, see Figure S4E-F for funnel plots.

### 3.5 Publication bias

Eggers’s test results were not significant for neither the pooled prevalence of all-cause dementia after performing the leave-one-out method (t = 1.60, *p* = 0.10), subgroups defined by sex (women: *t* = 1.26, *p* = 0.21; men: *t* = 1.18, *p* = 0.24), by area (rural: *t* = 0.50, *p* = 0.62; urban: *t* = 1.52, *p* = 0.13), or educational level (no formal education: *t* = 1.61, *p* = 0.11; at least one year of formal education: *t* = 2.01, *p* = 0.06), all suggesting absence of publication bias.

### 3.6 Qualitative findings

**Fig. 12:**
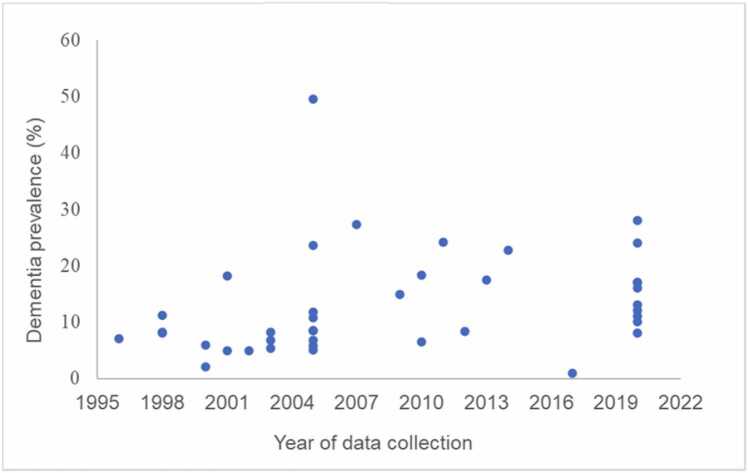
Dementia prevalence estimates across years of data collection.

We qualitatively verified that dementia prevalence was slightly higher in studies with more recent data collection, in the years between 2005 and 2020, compared to studies with data collection earlier than 2005 (Bottino et al., 2008, Brucki and Nitrini, 2014, Herrera et al., 2002, Ketzoian et al., 1997a; Llibre et al., 2009; Llibre Rodríguez et al., 1999; Lopes et al., 2012; Mejia-Arango and Gutierrez, 2011; Molero et al., 2007; Ramos-Cerqueira et al., 2005; Soto-Añari et al., 2021); however, robust assessment of secular increases in dementia prevalence was difficult considering the scarcity of data collected in the second half of the 2010 s. Estimates of all-cause dementia prevalence by year of data collection are represented in Fig. 12.

Studies had slight variations regarding the age inclusion criteria. Two studies included participants aged 50 + years (Brucki and Nitrini, 2014, Pedraza et al., 2017), one article included participants aged 55 + years (Molero et al., 2007). In contrast, 16 studies included participants aged 60 + years (Bartoloni et al., 2014, Bottino et al., 2008, César et al., 2016, Gatz et al., 2022, Gooding et al., 2006; Ketzoian et al., 1997a; Llibre Rodríguez et al., 1999; Lopes et al., 2012; Magalhães et al., 2008; Manrique-Espinoza et al., 2013, Mejia-Arango and Gutierrez, 2011, Mejia-Arango et al., 2021; Neita et al., 2014; Sanchez-Arenas et al., 2013; Soto-Añari et al., 2021), 11 included participants aged 65 + years (Albala et al., 1997; Correia et al., 2011, Custodio et al., 2008, Herrera et al., 2002, Llibre et al., 2009; Llibre Rodriguez et al., 2008; Martínez Querol et al., 2005; Ramos-Cerqueira et al., 2005; Scazufca et al., 2008; Villarreal et al., 2016), one included participants aged 70 + years (Davis et al., 2018) and one study included participants aged 75 + years (Caramelli et al., 2011). We thus report prevalence estimates by age group (50–59, 60–64, 65–69, 70–74, 75–79, 80 +) in Table 3. Some studies reported a slightly higher prevalence of dementia at earlier ages (> 64), going from 5.3 up to 44% (César et al., 2016, Gooding et al., 2006, Magalhães et al., 2008, Pedraza et al., 2017, Soto-Añari et al., 2021, Vargas-Alarcón et al., 2016) in comparison with the remaining studies, in which the percentage was around 2–3% at this age range. In Pedraza et al. (2017), the study was designed to include participants with 60 + years old; however, some 50–59 years-old adults were non-randomly selected into the study, resulting in a potentially biased estimate of dementia prevalence at ages 50–59.

**Table 3:** The number of study participants diagnosed with dementia against the total number of participants by age groups across the included studies.

Reference	Age group						
	50–59	60–64	65–69		70–74	75–79	≥ 80
Albala et al. (1997)	–	–	9.9%		18.3%	19.4%	52.4%
Bartoloni et al. (2014)	–	15/ 519 (2.7%)	24/435 (5.5%)		18/307 (5.8%)	13/200 (6.5%)	27/162 (16.7%)
Bottino et al. (2008)	–	9/368 (2.4%)	13/320 (4.1%)		22/311 (7.1%)	24/252 (9.5%)	39/226 (17.3%)
Brucki and Nitrini (2014)	–	–	–		–	–	–
Caramelli et al. (2011)	–	–	–		–	53/301 (17.61%)	122/337 (36.20%)
César et al. (2016)	–	8/152 (5.3%)	16/152 (10.5%)		18/117 (15.4%)	27/101 (26.7%)	41/108 (37.9%)
Correa Ribeiro et al. (2013)			3/79 (3.8%)		8/163 (4.9%)	14/158 (8.9%)	90/ 283 (31.8%)
Correia et al. (2011)	–	–	–		–	–	–
Custodio et al. (2008)	–	–	6/582 (1.0%)		9/ 428 (2.1%)	21/252 (8.4%)	67/270 (24.8%)
Davis et al. (2018)	–	–	–		81/676 (12%)	113/481 (23.5%)	248/675 (36.7%)
Eldemire- Shearer et al. (2018)	–	5/127 (3.9%)			12/116 (10.3%)		34/298 (11.4%)
Gatz et al. (2022)	–	203 / 0 (0%)		189 / 0 (0%)	110 / 0 (0%)	102/6 (5.80%)	
Gooding et al. (2006)	–	41/254^a^ (6.5%)			56/254 (8.4%)		56/135 (8.7%)
Herrera et al. (2002)	–	–	10/614 (1.6%)		15/470 (3.2%)	21/266 (7.9%)	72/306 (23.6%)
Ketzoian et al., 1997a^a^	–	–	–		–	–	–
Llibre et al. (2009)	–	–	–		171/5415 (3.2%)	179/4079 (4.4%)	947/8194 (11.6%)
Llibre Rodríguez et al. (1999)	–	–	–		–	–	–
Lopes et al. (2012)	–	5/269 (1.9%)	6/272 (2.2%)		15/251 (6.3%)	15/170 (9.7%)	28/163 (17.2%)
Llibre Rodriguez et al. (2008) (Cuba^b^)*	–	–	22/760 (2.9%)		47/789 (6.0%)	55/639 (8.6%)	191/749 (25.5%)
Llibre Rodriguez et al. (2008) (Dominican Republic^b^)*	–	–	21/532 (3.9%)		35/519 (6.7%)	50/397 (12.6%)	129/561 (23.0%)
Llibre Rodriguez et al. (2008) (Peru^b^)*	–	–	16/554 (2.9%)		17/493 (3.4%)	31/399 (7.8%)	100/486 (20.6%)
Llibre Rodriguez et al. (2008) (Venezuela^b^)*	–	–	19/812 (2.3%)		14/461 (3.0%)	19/340 (5.6%)	57/290 (19.7%)
Llibre Rodriguez et al. (2008) (Mexico^b^)*	–	–	5/544 (1.0%)		27/ 581 (4.6%)	39/347 (11.2%)	99/371 (26.7%)
Molero et al. (2007)	15/1074 (1.4%)		46/834 (5.5%)			135/530 (25.5%)	
Magalhães et al. (2008)	–	99/225 (44%)			78/148 (52.7%)		52/88 (59.1%)
Manrique-Espinoza et al. (2013)	–	–	–		–	–	–
Martínez Querol et al. 2005	–	–	–		–	–	–
Mejia-Arango and Gutierrez, 2011	–	–	–		–	–	–
Mejia-Arango et al. (2021)	–	233/4216 (5.5%)				518/2628 (19.7%)	
Neita et al. (2014)	–	0/40 (0%)	0/31 (0%)		1/36 (2.78%)	1/36 (2.78%)	11/57 (18.3%)
Pedraza et al. (2017)	12/185 (4.3%)	89/519 (31.8%)			111/400 (39.6%)		68/131 (24.3%)
Ramos-Cerqueira et al. (2005)	–	–	–		–	–	–
Sanchez-Arenas et al. (2013)		16/1495 (1.1%)			47/1053 (4.5%)		46/357 (12.9%)
Scazufca et al. (2008)	–	–	20/889 (2.2%)		11/556 (2.0%)	27/346 (7.8%)	47/281 (16.7%)
Soto-Añari et al. (2021)^c^		1531/ 147 (9.60%)	1417/ 176 (12.42%)		1045/151 (14.45%)	666/144 (21.62%)	586/200 (34.13%)
Villarreal et al. (2016)	–	–	–		–	–	–

*Note.*^a^based on age group 60–69. *Estimates based on diagnostic assessment with the 10/66 algorithm. ^b^If more than one sample included per study, samples further specified by country of data collection. ^c^ data available for the total study sample across countries.

## 4 Discussion

This systematic review and meta-analysis of the prevalence of all-cause dementia in LAC countries used strict inclusion criteria for dementia diagnosis and included research published in the predominant languages spoken in this region, resulting in an evidence based-comprehensive overview and providing updated and robust estimates of dementia prevalence for a region inhabited by around 8.4% of the world population.

This work strictly focused on dementia, not including cognitive impairment without dementia or mild cognitive impairment, especially because the latter does not present impairment in functionality, while the cognitive impairment in dementia is more severe and has a significant impact on activities of daily living (Gauthier et al., 2006). Although MCI patients have a higher risk of developing dementia, being considered by some authors as a prodromal stage of some dementias, reversion from MCI to being cognitively normal is also observed (Roberts et al., 2014). Therefore, we believe that considering a nosological and semiotic perspective, the two conditions should be considered as different disorders and hence our choice to exclude articles with no strict criteria for diagnosis of dementia. In addition, cognitive impairment is an impairment in one cognitive domain such as perception, orientation, attention, memory, learning, concentration, judgment or decision-making, intelligence, problem-solving, reaction-time, psychomotor ability, and social intactness (McDougall, 2017). Therefore, it can be a factor in a broader range of medical syndromes (Folstein et al., 1985). For this reason, the inclusion of articles with MCI or cognitive impairment would decrease the internal validity of this work and increase the prevalence of dementia. For a review of MCI prevalence in LAC, please see Ribeiro et al. (2021).

In our main analysis, we observed a prevalence of all-cause dementia of 10.66% for adults aged 50 or more. However, when excluding those articles detected as influencing the results by the leave-one-out method, the new prevalence estimate was 8.16%, including all the samples regardless of age range. Furthermore, we found a pooled prevalence of 7.43% for those studies including participants aged 50 + years and 8.96% for studies with participants aged 65 + years, the latter prevalence is slightly higher when compared with estimates from USA with 8.2%, which was also based on older participants aged 65 + years (Koller and Bynum, 2015), and higher than Europe with 7.1% (Bacigalupo et al., 2018) including participants with 55 + years of age, and much higher compared with estimates from China 5.3% in the population aged 60 + years (Yu-Tzu et al., 2018).

Low levels of education might explain the higher prevalence of dementia among older adults of LAC countries (Cao et al., 2020, Custodio et al., 2017, Mukadam et al., 2019), reflected in the higher number of illiteracy and low educational attainment across the selected studies, added to low socioeconomic levels, and limited access to primary health care and poor control of cardiovascular risk factors (Nitrini et al., 2020). In fact, the pooled analysis based on studies with rural samples showed a dementia prevalence of 8.68%, compared to 7.71% based on studies with urban samples. Moreover, not only the level of education but also lower-skilled occupations, which are predominantly manual, in the case of older adults in rural areas, are related to a higher prevalence of dementia (Alvares Pereira et al., 2021, Weden et al., 2018). However, in contrast to these studies, a recent study carried out by Gatz et al. (2022) found a very low (0.99%) prevalence of dementia in a representative sample of indigenous people in Bolivia. The authors suggest that this low prevalence of dementia is due to lower levels of cardiovascular risk factors and a physically active subsistence lifestyle. Nevertheless, Brucki and Nitrini (2014) observed an Amazonian population living in Sustainable Development Reserves with similar characteristics to Gatz et al. (2022) and found a 4.9% prevalence of dementia. These mixed findings suggest that more studies with rural and indigenous samples are necessary to comprehend specific factors leading to varied prevalence estimates in these populations, including assessments of the validity of dementia assessments in diverse populations with lower levels of formal schooling.

We observed a high heterogeneity between studies, possibly leading to the high variation in the crude prevalence, ranging from 0.99% to 49.57%. This disparity could be explained by several factors, such as samples coming from rural or urban regions, different socioeconomic backgrounds, ages, and also by the methods of diagnosing dementia, as the instruments and assessment strategies still broadly varied among studies – despite the rigorous inclusion criteria applied in this study. To arrive at more robust findings, it would be desirable to plan cross-national analyses through a consortium spanning different LAC countries and apply the same study protocol in order to reduce heterogeneity across studies.

Furthermore, as dementia is multifactorial in nature, unique factors in the LAC regions could have influenced this meta-analysis's results. For instance, dominantly inherited Alzheimer's disease pathogenic variant genes are detected among individuals living in LAC countries, with the highest frequencies in Colombia, Cuba, and Mexico (Llibre-Guerra et al., 2021). For other factors, however, there is little evidence to suggest that exposure-outcome relationships may differ in LAC countries. Findings similar to research in Western contexts have been found for variation in diet in LAC countries, for example, Albanese et al. (2009) tested the risk of dementia associated with fish and meat consumption in five LAC countries (Cuba, Dominican Republic, Mexico, Peru, and Venezuela), revealing that a higher fish intake was associated with lower prevalence of dementia. In this context, meat consumption was lower in Venezuela and highest in Dominican Republic, which is in congruence with the pooled all-cause dementia prevalence of this systematic review.

Moreover, other risk factors, such as physical exercise (Aarsland et al., 2010), could possibly be differentially protective in the LAC compared to Western contexts. However, current research assumes exposure-outcome relationships to be relatively uniform across contexts even if the prevalence of risk factors may be higher (Mukadam et al., 2019). In addition, air pollution may be a determinant influencing dementia prevalence, similarly to what research in Western contexts suggests. Finally, other potential determinants to be explored are the influence of weather and altitude on cognition, which only recently systematic investigations have begun, but which, however, also vary dramatically across LAC countries.

In a subsample of studies with sex-stratified estimates of dementia prevalence, we verified that dementia prevalence rate was higher among women than men. These findings seem to be explained by differences in higher life expectancy in women compared to men (Carter et al., 2012, Parra et al., 2018). Adding to this explanation, one should not minimise gender inequalities in education, work, and health, especially in low- and middle-income countries, meaning lower access to education, lower-income jobs, and lower access to health services by women (Carter et al., 2012, Snyder et al., 2016, Zurique Sánchez et al., 2019).

Given that high-income countries present a decrease in number of dementia prevalence lately (Langa et al., 2017, Matthews et al., 2013, Satizabal et al., 2016), we qualitatively investigated secular trends in LAC. Contrarily, our analysis showed an increase in dementia prevalence estimates in the studies with more recent data collection, which could be explained by increases in life expectancy in LAC, as there has been an increase of approximately seven years in life expectancy between 1990 and 2020 (United Nations, 2019). Although this meta-analysis did not explore healthy lifestyles due to the scarcity of data, it has been reported that LAC countries have experienced a change over the last 20 years to a detrimental lifestyle (FAO et al., 2021), specifically with increases in sedentary behaviour, poor eating habits, use of tobacco and alcohol, which play a crucial role in the increase of prevalence of chronic diseases such as cardiovascular disease, diabetes, and hypertension (OECD, 2020). In fact, a worse diet is associated with higher levels of cardiovascular risk factors, also leading to neurodegenerative damage (Feigin et al., 2016). Further, factors related to an unhealthy lifestyle, i.e., high body mass index, high fasting plasma glucose, and high intake of sugar-sweetened beverages, are strongly linked to Alzheimer’s disease and other dementias (Global Burden Disease Study, 2019). Consistent with secular increases in dementia prevalence identified in this meta-analysis, Ribeiro et al. (2021) showed that in one of the LAC countries, in Brazil, respondents aged 60 years or more were more likely to report diabetes, hypertension, and overweight/obesity in 2015 compared to 2000–2010.

Regarding the educational level of participants of the studies included in this review, we quantitatively observed that older adults with no formal education presented a much higher prevalence of dementia in comparison with older adults with at least one year of formal education (21.37% vs. 9.88%), corroborating with previous studies showing the protective effect of education against dementia (Langa et al., 2017, Livingston et al., 2020, Mukadam et al., 2019). However, it was not possible to include all the studies in the pooled prevalence by education due to the lack of information provided in some studies. Nevertheless, it was possible to qualitatively observe that the samples were predominantly composed of adult illiterates or up to four years of schooling, reflecting the reality of older people living in most LAC countries and may underlie the high dementia prevalence found in this review. In fact, previous studies exploring the contribution of educational level to dementia suggested inverse associations between the number of years of education and the risk of developing dementia (Custodio et al., 2008, Langa et al., 2017, Nitrini et al., 2009). In line with this, several studies propose that education can promote an increase in cognitive reserve, i.e., increased brain adaptability to compensate neuropathological and vascular damage before dementia symptoms appear (Mondini et al., 2016, Prince et al., 2012).

Regarding the methodological quality of studies assessed using the JBI critical appraisal checklist for studies reporting prevalence data, most studies generally complied well with the different aspects of the checklist. However, some studies lacked information on recruitment of participants, standardisation of measurements or response rate. Additionally, some studies did not report confidence intervals when describing statistical results.

### 4.1 Limitations and strengths of this study

This study provides updated and stratified estimates of dementia prevalence, firstly, for the LAC countries as a world region with less evidence on the epidemiology of dementia compared to other regions, such as Europe and the United States. Further, synthesising evidence from multiple studies that results in the most comprehensive review of dementia prevalence in LAC countries to date, our findings provide some confidence on the robustness of the dementia prevalence estimates. In fact, dementia is a health condition for which data scarcity and limited data quality are hampering the provision of serious estimates worldwide (Launer, 2019). Another strength of this study is the inclusion of studies with clear diagnostic criteria for dementia, as well as studies published in all languages predominantly spoken in LAC countries.

One limitation of this systematic review was the heterogeneity of the included studies, which could also be related to idiosyncrasies among LAC countries, in addition to the small number of investigations on dementia prevalence published in LAC countries. Actually, we did not find studies performed in the following countries: Belize, El Salvador, Honduras, Nicaragua, Costa Rica, Antigua and Barbuda, Bahamas, Barbados, Dominica, Grenada, Haiti, Kitts and Nevis, Lucia, Vincent and the Grenadines, Paraguay, Guyana, and Suriname. Filling these gaps in evidence for a significant part of this world region could be included in the scientific research agenda of large public health funders. Specifically, future studies could consider following strict criteria for diagnosis of dementia, such as DSM or ICD criteria, having more than one phase to screen and assess participants, performing different analyses considering age, sex, education, socioeconomic status, and assessing possible comorbidities, such as cardiovascular risk factors.

Moreover, only three articles explored genetic or biomarkers of dementia in this systematic review. Therefore, future studies should apply standardised cognitive assessment together with biofluid and neuroimaging biomarkers, besides assessing dementia-related risk factors at a population level to strengthen the robustness of dementia assessment. In this way, it would be feasible to assess possible interactions and mechanisms influencing dementia onset for a more complete picture (Ganguli et al., 2018).

## 5 Conclusions

Providing robust and updated dementia prevalence estimates for the large world region of LAC countries, this systematic review shows that dementia prevalence differs in the LAC countries according to sex and area, similar to evidence from other world regions such as Europe and the United States, although the prevalence rate is higher in LAC. Our meta-analysis point to possible increases in dementia prevalence in LAC studies with more recent data collection, questioning if the secular decreases in dementia incidence and prevalence observed in high-income countries hold in all world regions and are a strong reminder of the public health action on dementia in LAC countries. Considering rapidly ageing populations in the LAC countries, providing more robust estimates for the countries missing in this review would help estimate the societal and economic burden of dementia more accurately and assist in public health planning.

The high and rising prevalence of dementia in LAC showed the importance of recognising dementia as a growing public health priority, especially considering that LAC countries have limited resources to deal with this burden. For this reason, there is an urgent need for dialogue with health policy makers to effectively develop well-structured long-term strategic action plans for dementia in this region (Parra et al., 2018, Parra et al., 2021, Zurique Sánchez et al., 2019). In this sense, to respond to the pressures of an ageing society, it would require training of multidisciplinary teams to provide early and broad access to clinical and cognitive evaluations based on scientific consensus among LAC countries, which would enable early diagnosis (Fang et al., 2020), besides providing access to affordable treatments for dementia. Furthermore, the strategic plan should also address the prevention of modifiable risk factors throughout the life course (Livingston et al., 2020; Mukadam et at, 2019), such as giving access to sufficient and healthy food and equitable access to high-quality public education. In addition, to the promotion of public health interventions, including lifestyle modification programs during the life course, may also be critical in maintaining good cognitive functioning (Jin et al., 2021).

## Data Availability

Data will be made available on request.

## References

[bib1] Aarsland D., Sardahaee F.S., Anderssen S., Ballard C., Alzheimer's Society Systematic Review group (2010). Is physical activity a potential preventive factor for vascular dementia? A systematic review. Aging Ment. Health.

[bib2] Agência Nacional de Saúde Suplementar. (2021). ANS TABNET. Retrieved 26 September 2021, from 〈http://www.ans.gov.br/anstabnet/〉.

[bib3] Albala, C., Quiroga, P., Klaassen, G., Rioseco, P., Pérez, H., & Calvo, C. (1997). Prevalence of dementia and cognitive impairment in Chile.(Abstr). *World Congress of Gerontology*.

[bib4] Albanese E., Dangour A.D., Uauy R., Acosta D., Guerra M., Guerra S.S., Huang Y., Jacob K.S., de Rodriguez J.L., Noriega L.H., Salas A., Sosa A.L., Sousa R.M., Williams J., Ferri C.P., Prince M.J. (2009). Dietary fish and meat intake and dementia in Latin America, China, and India: a 10/66 Dementia Research Group population-based study. Am. J. Clin. Nutr..

[bib5] Alvares Pereira G., Silva Nunes M.V., Alzola P., Contador I. (2021). Cognitive reserve and brain maintenance in aging and dementia: an integrative review. Appl. Neuropsychol. Adult.

[bib6] Bacigalupo I., Mayer F., Lacorte E., Di Pucchio A., Marzolini F., Canevelli M., Di Fiandra T., Vanacore N. (2018). A systematic review and meta-analysis on the prevalence of dementia in europe: estimates from the highest-quality studies adopting the DSM IV diagnostic criteria. J. Alzheimer’s Dis. JAD.

[bib7] Bartoloni L., Blatt G., Insua I., Furman M., González M.A., Hermann B., Kesselman M., Massautis A., Reinado A., Senas P., Yavitz C., Lejarraga H., Nunes F., Arizaga R.L., Allegri R.F., Bartoloni L., Blatt G., Insua I., Furman M., Allegri R.F. (2014). A population-based study of cognitive impairment in socially vulnerable adults in Argentina. The Matanza Riachuelo Study. Preliminary Results. Dement. Neuropsychol..

[bib8] Bottino C.M.C., Azevedo D., Tatsch M., Hototian S.R., Moscoso M.A., Folquitto J., Scalco A.Z., Bazzarella M.C., Lopes M.A., Litvoc J. (2008). Estimate of dementia prevalence in a community sample from São Paulo, Brazil. Dement. Geriatr. Cogn. Disord..

[bib9] Brodaty H., Seeher K., Gibson L. (2012). Dementia time to death: a systematic literature review on survival time and years of life lost in people with dementia. Int. Psychogeriatr..

[bib10] Brucki S.M.D., Nitrini R. (2014). Cognitive impairment in individuals with low educational level and homogeneous sociocultural background. Dement. Neuropsychol..

[bib11] Cao Q., Tan C.-C., Xu W., Hu H., Cao X.-P., Dong Q., Tan L., Yu J.-T. (2020). The prevalence of dementia: a systematic review and meta-analysis. J. Alzheimers Dis..

[bib12] Caramelli P., Barbosa M.T., Sakurai E., Dos Santos E.L., Beato R.G., Machado J.C.B., Guimarães H.C., Teixeira A.L., Pietà Study Group (2011). The Pietà study: Epidemiological investigation on successful brain aging in Caeté (MG), Brazil. Methods and baseline cohort characteristics. Arq. De. Neuro-Psiquiatr..

[bib13] Carter C.L., Resnick E.M., Mallampalli M., Kalbarczyk A. (2012). Sex and gender differences in alzheimer’s disease: recommendations for future research. J. Women’s Health.

[bib14] César K.G., Brucki S.M.D., Takada L.T., Nascimento L.F.C., Gomes C.M.S., Almeida M.C.S., Oliveira M.O., Porto F.H.G., Senaha M.L.H., Bahia V.S., Silva T.B.L., Ianof J.N., Spíndola L., Schmidt M.T., Jorge M.S., Vale P.H.F., Cecchini M.A., Cassimiro L., Soares R.T., Nitrini R. (2016). Prevalence of cognitive impairment without dementia and dementia in tremembé, Brazil. Alzheimer Dis. Assoc. Disord..

[bib15] Chan J., Harky A. (2020). The importance of risk of bias assessment in meta-analyses: does controlling heterogeneity suffice. Eur. J. Cardio-Thorac. Surg.: Off. J. Eur. Assoc. Cardio-Thorac. Surg..

[bib16] Correa Ribeiro P.C., de Souza Lopes C., Lourenço R.A. (2013). Prevalence of dementia in elderly clients of a private health care plan: a study of the FIBRA-RJ, Brazil. Dement. Geriatr. Cogn. Disord..

[bib17] Correia C.C., Lima F., Junqueira F., Campos M.S., Bastos O., Petribú K., Laks J., Galvin J.E. (2011). AD8-Brazil: cross-cultural validation of the ascertaining dementia interview in Portuguese. J. Alzheimer’s Dis.: JAD.

[bib18] Custodio N., Wheelock A., Thumala D., Slachevsky A. (2017). Dementia in Latin America: epidemiological Evidence and Implications for Public Policy. Front. Aging Neurosci..

[bib19] Custodio N., García A., Montesinos R., Escobar J., Bendezú L. (2008). Prevalencia de demencia en una población urbana de Lima-Perú: Estudio puerta a puerta. . Fac. Med..

[bib20] Davis G., Baboolal N., Rae A.M., Stewart R. (2018). Dementia prevalence in a population at high vascular risk: the trinidad national survey of ageing and cognition. BMJ Open.

[bib21] Deeks J.J., Higgins J.P.T., Altman D.G., Higgins J.P.T., Thomas J., Chandler J., Cumpston M., Li T., Page M.J., Welch V.A. (2022). Cochrane Handbook for Systematic Reviews of Interventions version 6.3 (updated February 2022).

[bib22] Eldemire-Shearer D., Johnson P., Gibson R., James K., Willie-Tyndale D. (2018). Dementia among older persons in Jamaica: Prevalence and policy implications. West Indian Med. J. Open.

[bib23] Fang E.F., Xie C., Schenkel J.A., Wu C., Long Q., Cui H., Woo J. (2020). A research agenda for ageing in China in the 21st century (2nd edition): Focusing on basic and translational research, long-term care, policy and social networks. Ageing Res. Rev..

[bib24] FAO, IFAD, PAHO, WFP, UNICEF (2021).

[bib25] Feigin V.L., Roth G.A., Naghavi M., Parmar P., Krishnamurthi R., Chugh S., Mensah G.A., Norrving B., Shiue I., Ng M., Estep K., Cercy K., Murray C.J.L., Forouzanfar M.H. (2016).

[bib26] Fleiss J.L. (1981).

[bib27] Folstein M., Anthony J.C., Parhad I., Duffy B., Gruenberg E.M. (1985). The meaning of cognitive impairment in the elderly. J. Am. Geriatr. Soc..

[bib28] Freeman M.F., Tukey J.W. (1950). Transformations related to the angular and the square root. Ann. Math. Stat..

[bib29] Ganguli M., Albanese E., Seshadri S., Bennett D.A., Lyketsos C., Kukull W.A., Skoog I., Hendrie H.C. (2018). Population neuroscience: dementia epidemiology serving precision medicine and population health. Alzheimer Dis. Assoc. Disord..

[bib30] Gatz M., Mack W.J., Chui H.C., Law E.M., Barisano G., Sutherland M.L., Sutherland J.D., Eid Rodriguez D., Quispe Gutierrez R., Copajira Adrian J., Bani Cuata J., Borenstein A.R., Walters E.E., Irimia A., Rowan C.J., Wann L.S., Allam A.H., Thompson R.C., Miyamoto M.I., Michalik D.E., Kaplan H. (2022). Prevalence of dementia and mild cognitive impairment in indigenous Bolivian forager-horticulturalists. Alzheimer'S. Dement. J. Alzheimer'S. Assoc..

[bib31] Gauthier S., Reisberg B., Zaudig M., Petersen R.C., Ritchie K., Broich K., Belleville S., Brodaty H., Bennett D., Chertkow H., Cummings J.L., de Leon M., Feldman H., Ganguli M., Hampel H., Scheltens P., Tierney M.C., Whitehouse P., Winblad B., International Psychogeriatric Association Expert Conference on mild cognitive impairment (2006). Mild cognitive impairment. Lancet (Lond., Engl. ).

[bib32] GBD 2016 Dementia Collaborators (2019). Global, regional, and national burden of Alzheimer’s disease and other dementias, 1990-2016: A systematic analysis for the Global Burden of Disease Study 2016. Lancet Neurol..

[bib33] Gomes C. (2007). Intergenerational Exchanges in Mexico: Types and Intensity of Support. Curr. Sociol..

[bib34] Gooding, M.P., Amaya, E., Parra, M., & Ríos, A.M. (2006). Prevalencia de las demencias en el municipio de Neiva 2003–2005. 22(3), 6.

[bib35] Herrera E., Caramelli P., Silveira A.S.B., Nitrini R. (2002). Epidemiologic survey of dementia in a community-dwelling Brazilian population. Alzheimer Dis. Assoc. Disord..

[bib36] Ibáñez A., Pina-Escudero S.D., Possin K.L., Quiroz Y.T., Peres F.A., Slachevsky A., Sosa A.L., Brucki S.M.D., Miller B.L. (2021). Dementia caregiving across Latin America and the Caribbean and brain health diplomacy. Lancet Healthy Longev..

[bib37] Jin X., He W., Zhang Y., Gong E., Niu Z., Ji J., Li Y., Zeng Y., Yan L.L. (2021). Association of APOE ε4 genotype and lifestyle with cognitive function among Chinese adults aged 80 years and older: a cross-sectional study. PLoS Med..

[bib38] Ketzoian C., Romero S., Dieguez E., Cairolo G., Rega Y., Caseres R., Chouza C. (1997). Prevalence of demential sindromes in a population of Uruguay. Study of “Villa del Cerro”. J. Neurol. Sci..

[bib39] Ketzoian C., Rega I., Caseres R., Dieguez E., Coirolo G., Scaramelli A., Salamano R., Caamaño J.L., Romero S., Carrasco L., Pizzarossa C., Chouza C. (1997). Estudio de la prevalencia de las principales enfermedades neurológicas en una población del Uruguay. La Prensa Medica Uruguaya.

[bib40] Koller D., Bynum J.P.W. (2015). Dementia in the USA: State variation in prevalence. J. Public Health.

[bib41] Langa K.M., Larson E.B., Crimmins E.M., Faul J.D., Levine D.A., Kabeto M.U., Weir D.R. (2017). A Comparison of the Prevalence of Dementia in the United States in 2000 and 2012. JAMA Intern. Med..

[bib42] Launer L.J. (2019). Statistics on the burden of dementia: need for stronger data. Lancet Neurol..

[bib43] Lin L., Xu C. (2020). Arcsine-based transformations for meta-analysis of proportions: Pros, cons, and alternatives. Health Sci. Rep..

[bib44] Livingston G., Huntley J., Sommerlad A., Ames D., Ballard C., Banerjee S., Brayne C., Burns A., Cohen-Mansfield J., Cooper C., Costafreda S.G., Dias A., Fox N., Gitlin L.N., Howard R., Kales H.C., Kivimäki M., Larson E.B., Ogunniyi A., Orgeta V., Mukadam N. (2020). Dementia prevention, intervention, and care: 2020 report of the Lancet Commission. Lancet.

[bib45] Llibre J., de J., Fernández Y., Marcheco B., Contreras N., López A.M., Otero M., Gil I., Guerra M., Bayarre H., García M. (2009). Prevalence of dementia and Alzheimer’s disease in a Havana municipality: a community-based study among elderly residents. MEDICC Rev..

[bib46] Llibre Rodriguez J.J., Ferri C.P., Acosta D., Guerra M., Huang Y., Jacob K.S., Krishnamoorthy E.S., Salas A., Sosa A.L., Acosta I., Dewey M.E., Gaona C., Jotheeswaran A.T., Li S., Rodriguez D., Rodriguez G., Kumar P.S., Valhuerdi A., Prince M., 10/66 Dementia Research Group (2008). Prevalence of dementia in Latin America, India, and China: a population-based cross-sectional survey. Lancet.

[bib47] Llibre Rodríguez J.J., Guerra Hernández M.A., Pérez Cruz H., Bayarre Vea H., Fernández Ramírez S., González Rodríguez M., Samper Noa J.A. (1999). Síndrome demencial y factores de riesgo en adultos mayores de 60 años residentes en La Habana. Rev. De. Neurol..

[bib48] Llibre-Guerra J.J., Li Y., Allegri R.F., Mendez P.C., Surace E.I., Llibre-Rodriguez J.J., Sosa A.L., Aláez-Verson C., Longoria E.M., Tellez A., Carrillo-Sánchez K., Flores-Lagunes L.L., Sánchez V., Takada L.T., Nitrini R., Ferreira-Frota N.A., Benevides-Lima J., Lopera F., Ramírez L., Jiménez-Velázquez I., Bateman R.J. (2021). Dominantly inherited Alzheimer's disease in Latin America: Genetic heterogeneity and clinical phenotypes. Alzheimer'S. Dement.: J. Alzheimer's. Assoc..

[bib49] Lopes M.A., Ferrioli E., Nakano E.Y., Litvoc J., Bottino C.M.C. (2012). High prevalence of dementia in a community-based survey of older people from Brazil: association with intellectual activity rather than education. J. Alzheimer’s Dis. JAD.

[bib50] Maestre G.E., Mena L.J., Melgarejo J.D., Aguirre-Acevedo D.C., Pino-Ramirez G., Urribarri M., Chacon I.J., Chavez C., Falque-Madrid L., Gaona C.A., Terwilliger J.D., Lee J.H., Scarmeas N. (2018). Incidence of dementia in elderly Latin Americans: results of the Maracaibo Aging Study. Alzheimers Dement..

[bib51] Magalhães M.O., de C., Peixoto J.M., de S., Frank M.H., Gomes I., Rodrigues B.M., Menezes C., Cardoso E., Carvalho F., Aras R., Melo A. (2008). Risk factors for dementia in a rural area of Northeastern Brazil. Arq. De. Neuro Psiquiatr..

[bib52] Manrique-Espinoza B., Salinas-Rodríguez A., Margarita Moreno-Tamayo K. (2013). Condiciones de salud y estado funcional de los adultos mayores en México. Salud Pública De. México.

[bib53] Martínez Querol C., Pérez Martínez V.T., Carballo Pérez M., Varona Herrera G. (2005). Estudio clínico epidemiológico del síndrome demencial. Rev. Cuba. De. Med. Gen. Integral.

[bib54] Matthews F.E., Arthur A., Barnes L.E., Bond J., Jagger C., Robinson L., Brayne C. (2013). A two-decade comparison of prevalence of dementia in individuals aged 65 years and older from three geographical areas of England: Results of the Cognitive Function and Ageing Study I and II. Lancet.

[bib55] McDougall G.J. (2017). Assessing and addressing cognitive impairment in the elderly: a look at the research into cognitive impairment. Am. Nurse Today.

[bib56] Mejia-Arango S., Gutierrez L.M. (2011). Prevalence and incidence rates of dementia and cognitive impairment no dementia in the Mexican population: data from the Mexican Health and Aging Study. J. Aging Health.

[bib57] Mejia-Arango S., Avila J., Downer B., Garcia M.A., Michaels-Obregon A., Saenz J.L., Samper-Ternent R., Wong R. (2021). Effect of demographic and health dynamics on cognitive status in Mexico between 2001 and 2015: evidence from the mexican health and aging study. Geriatrics.

[bib58] Moher D., Liberati A., Tetzlaff J., Altman D.G., PRISMA Group (2010). Preferred reporting items for systematic reviews and meta-analyses: the PRISMA statement. Int. J. Surg..

[bib59] Molero A.E., Pino-Ramirez G., Maestre G.E. (2007). High prevalence of dementia in a Caribbean population. Neuroepidemiology.

[bib60] Mondini S., Madella I., Zangrossi A., Bigolin A., Tomasi C., Michieletto M., Villani D., Di Giovanni G., Mapelli D. (2016). Cognitive reserve in Dementia: implications for cognitive training. Front. Aging Neurosci..

[bib61] Mukadam N., Sommerlad A., Huntley J., Livingston G. (2019). Population attributable fractions for risk factors for dementia in low-income and middle-income countries: an analysis using cross-sectional survey data. Lancet Glob. Health.

[bib62] Munn Z., Moola S., Lisy K., Riitano D., Tufanaru C. (2015). Methodological guidance for systematic reviews of observational epidemiological studies reporting prevalence and cumulative incidence data. Int. J. Evid. -Based Healthc..

[bib63] Neita S.M., Abel W.D., Eldemire-Shearer D., James K., Gibson R.C. (2014). The prevalence and associated demographic factors of dementia from a cross-sectional community survey in Kingston, Jamaica. Int. J. Geriatr. Psychiatry.

[bib64] Nitrini R., Barbosa M.T., Brucki S.M.D., Yassuda M.S., Caramelli P. (2020). Current trends and challenges on dementia management and research in Latin America. J. Glob. Health.

[bib65] Nitrini R., Bottino C.M.C., Albala C., Capuñay N.S.C., Ketzoian C., Llibre Rodriguez J.J., Maestre G.E., Ramos-Cerqueira A.T.A., Caramelli P. (2009). Prevalence of dementia in Latin America: a collaborative study of population-based cohorts. Int. Psychogeriatr..

[bib66] OECD The World Bank (2020).

[bib67] Parra M.A., Baez S., Allegri R., Nitrini R., Lopera F., Slachevsky A., Custodio N., Lira D., Piguet O., Kumfor F., Huepe D., Cogram P., Bak T., Manes F., Ibanez A. (2018). Dementia in Latin America: Assessing the present and envisioning the future. Neurology.

[bib68] Parra M.A., Baez S., Sedeño L., Gonzalez Campo C., Santamaría-García H., Aprahamian I., Bertolucci P.H., Bustin J., Camargos Bicalho M.A., Cano-Gutierrez C., Caramelli P., Chaves M.L.F., Cogram P., Beber B.C., Court F.A., de Souza L.C., Custodio N., Damian A., de la Cruz M., Ibanez A. (2021). Dementia in Latin America: paving the way toward a regional action plan. Alzheimer’s Dement. J. Alzheimer’s Assoc..

[bib69] Pedraza O.L., Montes A.M.S., Sierra F.A., Montalvo M.C., Muñoz Y., Díaz J.M., Lozano A., Piñeros C. (2017). Mild cognitive impairment (MCI) and dementia in a sample of adults in the city of Bogotá. Dement. Neuropsychol..

[bib70] Pelaez M., Martinez I. (2002). Equity and systems of intergenerational transfers in Latin America and the Caribbean. Rev. Panam. De. Salud Pública.

[bib71] Prince M., Bryce R., Albanese E., Wimo A., Ribeiro W., Ferri C.P. (2013). The global prevalence of dementia: a systematic review and metaanalysis. Alzheimers Dement..

[bib72] Prince M., Acosta D., Ferri C.P., Guerra M., Huang Y., Rodriguez J.J.L., Salas A., Sosa A.L., Williams J.D., Dewey M.E., Acosta I., Jotheeswaran A.T., Liu Z. (2012). Dementia incidence and mortality in middle-income countries, and associations with indicators of cognitive reserve: a 10/66 Dementia Research Group population-based cohort study. Lancet.

[bib73] Prince M.J., Wu F., Guo Y., Gutierrez Robledo L.M., O’Donnell M., Sullivan R., Yusuf S. (2015). The burden of disease in older people and implications for health policy and practice. Lancet.

[bib74] Ramos-Cerqueira A.T.A., Torres A.R., Crepaldi A.L., Oliveira N.I.L., Scazufca M., Menezes P.R., Prince M. (2005). Identification of dementia cases in the community: a Brazilian experience. J. Am. Geriatr. Soc..

[bib75] Ribeiro F.S., de Oliveira Duarte Y.A., Santos J.L.F., Leist L.K. (2021). Changes in prevalence of cognitive impairment and associated risk factors 2000–2015 in São Paulo, Brazil. BMC Geriatr.

[bib76] Roberts R.O., Knopman D.S., Mielke M.M., Cha R.H., Pankratz V.S., Christianson T.J., Geda Y.E., Boeve B.F., Ivnik R.J., Tangalos E.G., Rocca W.A., Petersen R.C. (2014). Higher risk of progression to dementia in mild cognitive impairment cases who revert to normal. Neurology.

[bib77] Sanchez-Arenas R., Vargas-Alarcon G., Sanchez-Garcia S., Garcia-Peña C., Gutierrez-Gutierrez L., Grijalva I., Garcia-Dominguez A., Juárez-Cedillo T. (2013). Value of EQ-5D in Mexican city older population with and without dementia (SADEM study). Int. J. Geriatr. Psychiatry.

[bib78] Satizabal C.L., Beiser A.S., Chouraki V., Chêne G., Dufouil C., Seshadri S. (2016). Incidence of dementia over three decades in the framingham heart study. New Engl. J. Med..

[bib79] Scazufca M., Menezes P.R., Vallada H.P., Crepaldi A.L., Pastor-Valero M., Coutinho L.M.S., Di Rienzo V.D., Almeida O.P. (2008). High prevalence of dementia among older adults from poor socioeconomic backgrounds in Sao Paulo, Brazil. Int. Psychogeriatr..

[bib80] Shadish W.R., Haddock C.K., Cooper H., Hedges L.V. (1994). The Handbook of Research Synthesis.

[bib81] Snyder H.M., Asthana S., Bain L., Brinton R., Craft S., Dubal D.B., Espeland M.A., Gatz M., Mielke M.M., Raber J., Rapp P.R., Yaffe K., Carrillo M.C. (2016). Sex biology contributions to vulnerability to Alzheimer’s disease: a think tank convened by the Women’s Alzheimer’s Research Initiative. Alzheimer’s Dement.: J. Alzheimer’s Assoc..

[bib82] Soto-Añari M., Camargo L., Ramos-Henderson M., Rivera-Fernández C., Denegri-Solís L., Calle U., Mori N., Ocampo-Barbá N., López F., Porto M., Caldichoury-Obando N., Saldías C., Gargiulo P., Castellanos C., Shelach-Bellido S., López N. (2021). Prevalence of dementia and associated factors among older adults in Latin America during the COVID-19 pandemic. Dement. Geriatr. Cogn. Disord. Extra.

[bib83] StataCorp. 2021. Stata Statistical Software: Release 17. College Station, TX: StataCorp LLC.

[bib84] UNESCO (2017). https://bangkok.unesco.org/content/literacy-rates-rise-one-generation-next-challenges-remain-region.

[bib85] United Nations. (2019). *World Population Prospects—Population Division—United Nations*. 〈https://population.un.org/wpp/〉.

[bib86] United Nations Data Retrieval System (n.d.). Retrieved 12 August 2021, from 〈http://data.un.org/default.aspx〉.

[bib87] Vargas-Alarcón G., Juárez-Cedillo E., Martínez-Rodríguez N., Fragoso J.M., García-Hernández N., Juárez-Cedillo T. (2016). Association of interleukin-10 polymorphisms with risk factors of Alzheimer’s disease and other dementias (SADEM study). Immunol. Lett..

[bib88] Villarreal A.E., Pérez-Lao A.R., Oviedo D.C., Grajales S., Carreira M.B., Britton G.B. (2019). The panama aging research initiative longitudinal study. MEDICC Rev..

[bib89] Villarreal A.E., Grajales S., O’Bryant S.E., Edwards M., López L., Montalván A., Britton G.B., Panama Aging Research Initiative (PARI) (2016). Characterization of Alzheimer’s disease and mild cognitive impairment in older adults in panama. J. Alzheimer’s Dis. JAD.

[bib90] Weden M.M., Shih R.A., Kabeto M.U., Langa K.M. (2018). Secular trends in dementia and cognitive impairment of U.S. rural and urban older adults. Am. J. Prev. Med..

[bib91] World Health Organization. (2020). *Dementia.* 〈https://www.who.int/news-room/fact-sheets/detail/dementia〉.

[bib92] Xiang Y., Vilmenay K., Poon A.N., Ayanian S., Aitken C.F., Chan K.Y. (2021). Systematic review estimating the burden of dementia in the Latin America and caribbean region: a bayesian approach. Front. Neurol..

[bib93] Yu-Tzu Wu, Gemma-Claire Ali, Maëlenn Guerchet, Matthew Prina A., Kit Yee Chan, Martin Prince, Carol Brayne (2018). Prevalence of dementia in mainland China, Hong Kong and Taiwan: an updated systematic review and meta-analysis. Int. J. Epidemiol..

[bib94] Zurique Sánchez C., Cadena Sanabria M.O., Zurique Sánchez M., Camacho López P.A., Sánchez Sanabria M., Hernández Hernández S., Velásquez Vanegas K., Ustate Valera A. (2019). Prevalencia de demencia en adultos mayores de América Latina: revisión sistemática. Rev. Esp. De. Geriatría Y. Gerontol..

