# Supplementary material for: Prevalence of dementia in Latin America and Caribbean countries: Systematic review and meta-analyses exploring age, sex, rurality, and education as possible determinants
Source: Ageing Res Rev. 2022 Nov;81:101703. doi: 10.1016/j.arr.2022.101703 (PMC9582196; doi:10.1016/j.arr.2022.101703)
Supplement: Supplementary file 1 — Supplementary material. [file mmc1.docx]

**Supplementary Material**

The search terms for each database and number of found articles

**Updated search on 13 – 04– 2022**

**Pubmed (n = 1063)**

(dementia [MeSH]) AND (Prevalence OR Epidemiology) AND ("Latin America" OR “South America” OR Caribbean OR Argentina OR Bolivia OR Brazil OR Chile OR Colombia OR "Costa Rica" OR Cuba OR Ecuador OR "El Salvador" OR Guatemala OR Haiti OR Honduras OR Mexico OR Nicaragua OR Panama OR Paraguay OR Peru OR "Dominican Republic" OR Uruguay OR Venezuela OR Jamaica OR “Trinidad and Tobago” OR Guyana OR Suriname OR Belize OR Bahamas OR Barbados OR “Saint Lucia” OR Grenada OR “St. Vincent and Grenadines” OR “Antigua and Barbuda” OR Dominica OR “Saint Kitts and Nevis”)

**Web of knowledge (n = 1273)**

(Dementia) AND (Prevalence OR Epidemiology) AND ("Latin America" OR “South America” OR Caribbean OR Argentina OR Bolivia OR Brazil OR Chile OR Colombia OR "Costa Rica" OR Cuba OR Ecuador OR "El Salvador" OR Guatemala OR Haiti OR Honduras OR Mexico OR Nicaragua OR Panama OR Paraguay OR Peru OR "Dominican Republic" OR Uruguay OR Venezuela OR Jamaica OR “Trinidad and Tobago” OR Guyana or Suriname OR Belize OR Bahamas OR Barbados OR “Saint Lucia” OR Grenada OR “St. Vincent and Grenadines” OR “Antigua and Barbuda” OR Dominica OR “Saint Kitts and Nevis”)

**Scopus (n = 579)**

TITLE-ABS-KEY ( ( ( dementia ) AND ( prevalence OR epidemiology ) AND ( "Latin America" OR "South America" OR caribbean OR argentina OR bolivia OR brazil OR chile OR colombia OR "Costa Rica" OR cuba OR ecuador OR "El Salvador" OR guatemala OR haiti OR honduras OR mexico OR nicaragua OR panama OR paraguay OR peru OR "Dominican Republic" OR uruguay OR venezuela OR jamaica OR "Trinidad and Tobago" OR guyana OR suriname OR belize OR bahamas OR barbados OR "Saint Lucia" OR grenada OR "St. Vincent and Grenadines" OR "Antigua and Barbuda" OR dominica OR "Saint Kitts and Nevis" ) ) )

**Lilacs - (n= 1359)**

mh:((dementia) OR (demencia) OR (demência) AND (prevalenc*) OR (prevalência) OR (epidemiolog*)) AND ( db:("LILACS"))

**SciELO (n= 88)**

( dementia ) AND ( prevalence OR epidemiology ) AND ( "Latin America" OR "South America" OR caribbean OR argentina OR bolivia OR brazil OR chile OR colombia OR "Costa Rica" OR cuba OR ecuador OR "El Salvador" OR guatemala OR haiti OR honduras OR mexico OR nicaragua OR panama OR paraguay OR peru OR "Dominican Republic" OR uruguay OR venezuela OR jamaica OR "Trinidad and Tobago" OR guyana OR suriname OR belize OR bahamas OR barbados OR "Saint Lucia" OR grenada OR "St. Vincent and Grenadines" OR "Antigua and Barbuda" OR dominica OR "Saint Kitts and Nevis" )

Table S1. Excluded articles and reasons for their exclusion.

| **Author** | **Publication Year** | **Study** | **Reasons for exclusion** |
| --- | --- | --- | --- |
| Ferri, C.P., & Prince, M. | 2010 | 10/66 Dementia Research Group: recently published survey data for seven Latin America sites | Non-original data |
| Prince, M.J., et al. | 2008 | The 10/66 Dementia Research Group's fully operationalised DSM-IV dementia computerized diagnostic algorithm, compared with the 10/66 dementia algorithm and a clinician diagnosis: a population validation study | Non-original data |
| Acosta, D., et al. | 2021 | Dementia Research in the Caribbean Hispanic Islands: Present Findings and Future Trends | Non-original data |
| Aarsland, V., et al. | 2020 | Association between physical activity and cognition in Mexican and Korean older adults | Non-original data |
| Acosta, D., et al. | 2008 | The epidemiology of dependency among urban-dwelling older people in the Dominican Republic; a cross-sectional survey | Non-original data |
| Acosta, D., et al. | 2010 | The prevalence and social patterning of chronic diseases among older people in a population undergoing health transition. A 10/66 Group cross-sectional population-based survey in the Dominican Republic | Non-original data |
| Acosta, I., et al. | 2018 | Neuropsychiatric symptoms as risk factors of dementia in a Mexican population: A 10/66 Dementia Research Group study | Non-original data |
| Albanese, E. et al. | 2009 | Dietary fish and meat intake and dementia in Latin America, China, and India: a 10/66 Dementia Research Group population-based study | Non-original data |
| Albanese, E., et al. | 2013 | Dementia and lower blood pressure in Latin America, India, and China: a 10/66 cross-cohort study | Non-original data |
| Albanese, E., et al. | 2013 | Dementia severity and weight loss: a comparison across eight cohorts. The 10/66 study | Non-original data |
| Avila, R., et al. | 2016 | Normative data of Fuld Object Memory Evaluation test for brazilian elderly population | Non-original data |
| Bae, J.B., et al. | 2020 | Does parity matter in women's risk of dementia? A COSMIC collaboration cohort study | Non-original data |
| Bao J., et al. | 2019 | Multimorbidity and care dependence in older adults: a longitudinal analysis of findings from the 10/66 study | Non-original data |
| Bensenor, I.M., et al. | 2008 | Anemia and dementia among elderly: The Sao Paulo ageing and health (spah) study, Brazil | Non-original data |
| Bottino, C.M.C., et al. | 2005 | Prevalence of dementia and MCI in Sao Paulo, Brazil | Non-original data |
| Caramelli, P., Barbosa, M.T., Sakurai, E., dos Santos, E.L., Beato, R.G., Machado, J.C.B., Guimar̃es, H.C., Teixeira, A.L. | 2011 | Epidemiological investigation on successful brain aging in caeté (MG), brazil: Methods and baseline cohort characteristics | Non-original data |
| César, K. G. | 2014 | Prevalence study of mild cognitive impairment and dementia in Tremembé city, São Paulo state | Non-original data |
| César, K.G., et al. | 2017 | Addenbrooke's cognitive examination-revised: normative and accuracy data for seniors with heterogeneous educational level in Brazil | Non-original data |
| Daskalopoulou, C. et al. | 2019 | Development of a healthy ageing index in Latin American countries - a 10/66 dementia research group population-based study | Non-original data |
| Daskalopoulou, C., et al. | 2019 | Healthy ageing and the prediction of mortality and incidence dependence in low- and middle- income countries: a 10/66 population-based cohort study | Non-original data |
| Daskalopoulou, C., et al. | 2018 | Associations of Lifestyle Behaviour and Healthy Ageing in Five Latin American and the Caribbean Countries-A 10/66 Population-Based Cohort Study | Non-original data |
| Davis, G., et al. | 2021 | Health status risk factors and quality of life in 75-84-year-old individuals assessed for dementia using the short 10/66 dementia diagnostic schedule | Non-original data |
| Gil, M., et al. | 2021 | Neuropsychiatric Symptoms Among Hispanics: Results of the Maracaibo Aging Study | Non-original data |
| Herrera Junior, E. et al. | 1998 | Population epidemiologic study of dementia in Catanduva city: state of Sao Paulo, Brazil | Non-original data |
| Honyashiki, M., et al. | 2011 | Chronic diseases among older people and co-resident psychological morbidity: a 10/66 Dementia Research Group population-based survey | Non-original data |
| Hototian, S.R., et al. | 2008 | Prevalence of cognitive and functional impairment in a community sample from Sao Paulo, Brazil | Non-original data |
| Hototian, S.R., et al. | 2004 | Dementia prevalence study in a community sample of Sao Paulo, Brazil | Non-original data |
| Johansson, L. et al. | 2019 | Associations between Depression, Depressive Symptoms, and Incidence of Dementia in Latin America: A 10/66 Dementia Research Group Study | Non-original data |
| Kawabata-Yoshihara, L.A. et al. | 2012 | Atrial Fibrillation and Dementia: Results from the Sao Paulo Ageing & Health Study | Non-original data |
| Li, J.,et al. | 2021 | Associations between education and dementia in the caribbean and the United States: An international comparison | Non-original data |
| Lipnicki, D.M. et al. | 2017 | Age-related cognitive decline and associations with sex, education and apolipoprotein E genotype across ethnocultural groups and geographic regions: a collaborative cohort study | Non-original data |
| Lipnicki, D.M., et al. | 2019 | Determinants of cognitive performance and decline in 20 diverse ethno-regional groups: A COSMIC collaboration cohort study | Non-original data |
| Llibre Rodríguez, J., & Gutiérrez Herrera, R.F. | 2014 | Dementias and Alzheimer's disease in Latin America and the Caribbean | Non-original data |
| Llibre Rodríguez, J., et al. | 2008 | The prevalence, correlates and impact of dementia in Cuba: A 10/66 group population-based survey | Non-original data |
| Llibre, J. J., et al. | 2011 | Dementia and other chronic diseases in older adults in Havana and Matanzas: the 10/66 study in Cuba | Non-original data |
| Llibre-Guerra, J.C., et al. | 2015 | Stroke incidence and risk factors in Havana and Matanzas, Cuba | Non-original data |
| Llibre-Guerra, J.J., et al. | 2016 | Prevalence, incidence and associations between apoe genotype, cardiovascular risk factor and dementia in the Cuban population | Non-original data |
| Llibre-Guerra, J.J., et al. | 2022 | Race, Genetic Admixture, and Cognitive Performance in the Cuban Population | Non-original data |
| Llibre-Rodríguez, J.J., et al. | 2017 | Cuba's Aging and Alzheimer Longitudinal Study | Non-original data |
| Lopes, M. A. | 2006 | Epidemiological survey of prevalence of dementia in RibeirÃ£o Preto | Non-original data |
| Lopes, M. A., & Bottino, C. M. C | 2002 | Prevalence of dementia in several regions of the world: analysis of epidemiologic studies from 1994 to 2000 | Non-original data |
| Lopes, M.A., et al. | 2010 | Prevalence of alcohol-related problems in an elderly population and their association with cognitive impairment and dementia | Non-original data |
| Lopes, M.A., et al. | 2005 | Prevalence of dementia and Alzheimer's disease in Ribeirao Preto, Brazil: A community survey in elderly population | Non-original data |
| Lopes, M.A., et al. | 2004 | Dementia prevalence in a community sample from Ribeirao Preto, Brazil | Non-original data |
| Lourenco, R. A., & Sanchez, M.A.D. | 2014 | Accuracy of the Brazilian Version of the Informant Questionnaire on Cognitive Decline in the Elderly at Screening for Dementia in Community-Dwelling Elderly Participants: Findings From FIBRA-RJ Study | Non-original data |
| Maestre, G.E., et al. | 2018 | Incidence of dementia in elderly Latin Americans: Results of the Maracaibo Aging Study | Non-original data |
| Mayston, R., et al. | 2014 | Exploring the economic and social effects of care dependence in later life: protocol for the 10/66 research group INDEP study | Non-original data |
| Molero, A.E., Pino-Ramírez, G, Maestre, GE. | 2007 | High prevalence of dementia in a Caribbean population | Non-original data |
| Mukadam, N., et al. | 2019 | Population attributable fractions for risk factors for dementia in low-income and middle-income countries: an analysis using cross-sectional survey data | Non-original data |
| Peeters, G., et al. | 2020 | Risk Factors for Incident Dementia Among Older Cubans | Non-original data |
| Perales-Puchalt J., et al. | 2019 | Cardiovascular health and dementia incidence among older adults in Latin America: Results from the 10/66 study | Non-original data |
| Prina, A.M., et al. | 2017 | Cohort Profile: The 10/66 study | Non-original data |
| Prince, M., et al. | 2003 | Dementia diagnosis in developing countries: a cross-cultural validation study | Non-original data |
| Prince, M., et al. | 2012 | Dementia incidence and mortality in middle-income countries, and associations with indicators of cognitive reserve: a 10/66 Dementia Research Group population-based cohort study | Non-original data |
| Rodríguez, J.J.L., et al. | 2014 | Incidence of dementia and association with apoe genotype in older Cubans | Non-original data |
| Rodriguez-Agudelo, Y. et al. | 2011 | Neuropsychiatric symptoms in older adults with and without dementia in urban and rural regions. Results of the 10/66 Dementia Research Group in Mexico | Non-original data |
| Russo, M.J., et al. | 2017 | High Prevalence of Mild Cognitive Impairment and Dementia in a social vulnerable population in Argentina: A starting point for dementia prevention. | Non-original data |
| Scazufca, M. et al. | 2009 | Limitations of the Mini-Mental State Examination for screening dementia in a community with low socioeconomic status | Non-original data |
| Scazufca, M., et al. | 2005 | Prevalence of dementia among low income elderly in a district of Sao Paulo, Brazil | Non-original data |
| Scazufca, M., et al. | 2010 | The role of literacy, occupation and income in dementia prevention: the Sao Paulo Ageing & Health Study (SPAH) | Non-original data |
| Scazufca, M., et al. | 2008 | High prevalence of dementia among older adults from poor socioeconomic backgrounds in São Paulo, Brazil | Non-original data |
| Sousa, R.M., et al. | 2009 | Contribution of chronic diseases to disability in elderly people in countries with low and middle incomes: a 10/66 Dementia Research Group population-based survey | Non-original data |
| Stephan, B.C.M., et al. | 2020 | Prediction of dementia risk in low-income and middle-income countries (the 10/66 Study): an independent external validation of existing models | Non-original data |
| Stewart, R. et al. | 2016 | Development of a brief assessment and algorithm for ascertaining dementia in low-income and middle-income countries: the 10/66 short dementia diagnostic schedule | Non-original data |
| Tatsch, M.F., et al. | 2006 | Neuropsychiatric symptoms in Alzheimer disease and cognitively impaired, nondemented elderly from a community-based sample in Brazil: prevalence and relationship with dementia severity | Non-original data |
| Teruel, B.M., et al. | 2011 | Interactions between genetic admixture, ethnic identity, APOE genotype and dementia prevalence in an admixed Cuban sample; a cross-sectional population survey and nested case-control study | Non-original data |
| Vargas-Alarcón, G., et al. | 2016 | Association of interleukin-10 polymorphisms with risk factors of Alzheimer's disease and other dementias (SADEM study) | Non-original data |
| Yeverino-Castro, S.G. et al. | 2021 | Prevalence and incidence of possible vascular dementia among Mexican older adults: Analysis of the Mexican Health and Aging Study | Non-original data |
| Custodio, N. et al | 2003 | Prevalence of vascular dementia in a longitudinal study | Not found |
| Galli Silva, E. | 1995 | Epidemiology of Alzheimer's disease | Not found |
| Gomes, M.M. | 1995 | Epidemiology of demential disorders | Not found |
| LozanoAscencio, R. et al. | 1996 | Burden of disease in the elderly, Mexico 1994 | Not found |
| Morales Virgen, J. J. | 1997 | Epidemiology in dementias | Not found |
| Nitrini, R., et al. | 2004 | Incidence of dementia in a community-dwelling Brazilian population | Not found |
| Perez, O., et al. | 1993 | Prevalence of dementia in La Habana [PREVALENCIA DE DEMENCIA EN LA HABANA] | Not found |
| Rodriguez, J.L. | 2002 | Epidemiology of dementia and Alzheimer's disease in a population over 65 years in Cuba | Not found |
| Aliberti, M.J.R., et al. | 2019 | Assessing Risk for Adverse Outcomes in Older Adults: The Need to Include Both Physical Frailty and Cognition | Studies were not from LAC |
| Arce Rentería, M., et al. | 2019 | Illiteracy, dementia risk, and cognitive trajectories among older adults with low education | Studies were not from LAC |
| Crisostomo, P.R.,et al. | 2002 | Prevalence of cognitive impairment in an urban Hispanic community population [5] | Studies were not from LAC |
| Dos Santos, C.S., et al. | 2020 | Factors associated with dementia in elderly [Fatores associados à demência em idosos] | Studies were not from LAC |
| Farfel, J.M. et al. | 2019 | Alzheimer's disease frequency peaks in the tenth decade and is lower afterwards | Studies were not from LAC |
| Feter, N., et al. | 2021 | Physical activity attenuates the risk for dementia associated with aging in older adults with mild cognitive impairment. Findings from a population-based cohort study | Studies were not from LAC |
| Feter, N., et al. | 2021 | Physical activity in later life and risk of dementia: Findings from a population-based cohort study | Studies were not from LAC |
| Haan, M.N., et al. | 2003 | Prevalence of dementia in older latinos: the influence of type 2 diabetes mellitus, stroke and genetic factors | Studies were not from LAC |
| McCracken, C.F., et al. | 1997 | Prevalence of dementia and depression among elderly people in black and ethnic minorities | Studies were not from LAC |
| Paul, K.C. et al. | 2018 | Cognitive decline, mortality, and organophosphorus exposure in aging Mexican Americans | Studies were not from LAC |
| Robson, J., et al. | 2021 | NHS Health Checks: an observational study of equity and outcomes 2009-2017 | Studies were not from LAC |
| Ryan, B., et al. | 2021 | Sociodemographic and Clinical Characteristics of 1350 Patients With Young Onset Dementia A Comparison With Older Patients | Studies were not from LAC |
| Lini, E. V., et al. | 2016 | Prevalence and factors associated with symptoms suggestive of dementia in the elderly | Studies were not from LAC e CI |
| Yanez, N.et al. | 2020 | Analyses of Mortality and Prevalence of Cerebrovascular Disease in Colombia, South America (2014-2016): A Cross-Sectional and Ecological Study | Not focused in dementia in older population |
| Aliberti, M., et al. | 2021 | Frailty modifies the association of hypertension with cognition in older adults: Evidence from the elsi-Brazil | Not focused in dementia prevalence |
| Feter, N., et al. |  | Are gender differences in physical inactivity associated with the burden of dementia in low- and lower-middle income countries? | Not focused in dementia prevalence |
| Maestre, G.E., et al. | 2002 | The Maracaibo Aging Study: population and methodological issues | Not focused on dementia |
| Ketzoian, C. | 1994 | The prevalence of leading neurologic diseases in Uruguay: a pilot study | Not related to dementia prevalence |
| Sariya, S., et al. | 2021 | Polygenic Risk Score for Alzheimer's Disease in Caribbean Hispanics | Not related to dementia prevalence |
| César-Freitas, K.G., et al. | 2021 | Incidence of dementia in a Brazilian population: The Tremembé Epidemiologic Study | Not related to dementia prevalence |
| Pérez Akly, M., et al. | 2017 | Multicentric epidemiological study in amyotrophic lateral sclerosis in the Autonomous City of Buenos Aires | Not related to dementia prevalence |
| Quevedo, A.L.A., et al. | 2017 | Analysis of prevalence of self-reported hearing loss and associated factors: primary versus proxy informant | Not related to dementia prevalence |
| Sánchez López, M., et al. | 1992 | Depression prevalence in non-institutionalized | Not related to dementia prevalence |
| Santos, I.S. | 2009 | Anemia prevalence in older subjects, causes of persistence or recurrence and its relation with dementia | Not related to dementia prevalence/ Non-original data |
| Ashby-Mitchell, K., et al. | 2020 | Proportion of Dementia Explained by Five Key Factors in Jamaica | Not focused in dementia prevalence |
| Santos, I.S., et al. | 2012 | Anemia and dementia among the elderly: the Sao Paulo Ageing & Health Study | Not related to dementia prevalence/Non-original data |
| Nitrini, R., et al. | 2009 | Prevalence of dementia in Latin America: a collaborative study of population-based cohorts | Theoretical, protocol, editorial, or reviews |
| Arizaga, R.L., et al. | 1999 | Vascular dementia: the Latin American perspective | Theoretical, protocol, editorial, or reviews |
| Baboolal, N., et al. | 2014 | Trinidad and tobago :A decade of dementia research | Theoretical, protocol, editorial, or reviews |
| Brito-Aguilar, R. | 2019 | Dementia around the World and the Latin America and Mexican Scenarios | Theoretical, protocol, editorial, or reviews |
| Burlá, C. et al. | 2013 | A perspective overview of dementia in Brazil: a demographic approach | Theoretical, protocol, editorial, or reviews |
| Del Brutto, O.H., et al | 2003 | Door-to-door survey of neurological diseases in Atahualpa, a rural village in the ecuadorian coastal region - Methodology and operational definitions | Theoretical, protocol, editorial, or reviews |
| Del Brutto, O.H., et al. | 2020 | Cerebrovascular Correlates of Dementia in Community- Dwelling Older Adults Living in Rural Communities - The Three Villages Study. Rationale and Protocol of a Population- Based Prospective Cohort Study | Theoretical, protocol, editorial, or reviews |
| Ferri, C.P. | 2012 | Population ageing in Latin America: dementia and related disorders | Theoretical, protocol, editorial, or reviews |
| Kalaria, R.N. | 2008 | Alzheimer's disease and vascular dementia in developing countries: prevalence, management, and risk factors | Theoretical, protocol, editorial, or reviews |
| Nitrini, R. | 1999 | Epidemiology of Alzheimer's disease in Brazil | Theoretical, protocol, editorial, or reviews |
| Prince, M. et al. | 2004 | Alzheimer Disease International's 10/66 Dementia Research Group - one model for action research in developing countries | Theoretical, protocol, editorial, or reviews |
| Rowe, J.S. | 2014 | Alzheimer's disease and other dementia in sub-Saharan Africa and the Caribbean | Theoretical, protocol, editorial, or reviews |
| Scazufca, M., et al. | 2002 | Epidemiological research on dementia in developing countries | Theoretical, protocol, editorial, or reviews |
| Sosa-Ortiz, A.L., et al. | 2012 | Epidemiology of dementias and Alzheimer's disease | Theoretical, protocol, editorial, or reviews |
| Toledo, A. A. S. F. et al. | 2014 | Brazilian research on cognitive impairment and dementia from 1999 to 2013 | Theoretical, protocol, editorial, or reviews |
| Vargas, E.A., et al. | 2014 | Prevalence of dementia incolombian populations | Theoretical, protocol, editorial, or reviews |
| Vega, I.E. et al. | 2017 | Alzheimer's Disease in the Latino Community: Intersection of Genetics and Social Determinants of Health | Theoretical, protocol, editorial, or reviews |
| Villarreal, A.E., et al. | 2019 | The Panama Aging Research Initiative Longitudinal Study | Theoretical, protocol, editorial, or reviews |
| Del Brutto, O.H., et al. | 2004 | Door-to-door survey of major neurological diseases in rural Ecuador--the Atahualpa Project: methodological aspects | protocol / Not focused in dementia prevalence |
| Atalaia-Silva, K. C. & Lourenço, R. A. | 2008 | Translation, adaptation and construct validation of the Clock Test among elderly in Brazil | Studies did not meet dementia |
| Pagés Larraya, F., et al. | 1999 | Prevalence of the Alzheimer type dementia in Buenos Aires City | Studies did not meet dementia |
| Pradilla, G., et al | 2002 | Neuro-epidemiology in the eastern region of Colombia | Studies did not meet dementia |
| Ramos, L.R., et al. | 1998 | Two-year follow-up study of elderly residents in S Paulo, Brazil: methodology and preliminary results | Studies did not meet dementia |
| Velázquez-Brizuela, I.E., Ortiz, G.G., Ventura-Castro, L., Árias-Merino, E.D., Pacheco-Moisés, F.P., Macías-Islas, M.A. | 2014 | Prevalence of dementia, emotional state and physical performance among older adults in the metropolitan area of guadalajara, Jalisco, Mexico | Studies did not meet dementia |
| Viana, G. S. B., et al. | 1991 | Aplicação do teste de informação, memória e concentração (IMC) ao estudo epidemiológico de demência senil em Fortaleza | Studies did not meet dementia |
| Andrade, F.C.D., et al. | 2014 | Life expectancy with and without cognitive impairment among Brazilian older adults | Studies did not meet dementia criteria |
| AnzolaPerez, E., et al. | 1996 | Towards community diagnosis of dementia: Testing cognitive impairment in older persons in Argentina, Chile and Cuba | Studies did not meet dementia criteria |
| Arizaga, R. L., et al. | 2014 | Cognitive impairment and risk factor prevalence in a population over 60 in Argentina | Studies did not meet dementia criteria |
| Arizaga, R.L., et al. | 2005 | Cognitive impairment population based study in people 60 and over in Cañuelas (Argentina). Results from the Pilot "Ceibo Study" | Studies did not meet dementia criteria |
| Ashby-Mitchell, K. et al. | 2015 | Life Expectancy with and without Cognitive Impairment in Seven Latin American and Caribbean Countries | Studies did not meet dementia criteria |
| Barcelos-Ferreira, R., et al. | 2009 | Clinically Significant Depressive Symptoms and Associated Factors in Community Elderly Subjects From Sao Paulo, Brazil | Studies did not meet dementia criteria |
| Cabezas, R. D., et al. | 2013 | Prevalence of cognitive impairment and dementia in people older 65 years in a colombian urban population | Studies did not meet dementia criteria |
| Cabrera, M.A.S., et al. | 2016 | Cognitive and Functional Decline among Individuals 50 Years of Age or Older in Cambe, Parana, Brazil: A Population-Based Study | Studies did not meet dementia criteria |
| Caixeta, L., & Reis, G.S. | 2011 | DEMENTIA PREVALENCE IN INDIGENOUS POPULATION FROM BRAZILIAN AMAZON | Studies did not meet dementia criteria |
| Confortin, S.C., et al. | 2019 | Anthropometric indicators associated with dementia in the elderly from Florianopolis - SC, Brazil: EpiFloripa Ageing Study | Studies did not meet dementia criteria |
| del Pozo, P.H.E., et al. | 2011 | Risk Factors and Prevalence of Dementia and Alzheimer's Disease in Pichincha, Ecuador - FARYPDEA Study | Studies did not meet dementia criteria |
| del Pozo, P.H.E., et al. | 2020 | Detecting Cognitive Decline and Dementia in Santa Cruz, Galapagos Islands, Ecuador | Studies did not meet dementia criteria |
| del Pozo, P.H.E., et al. | 2018 | Cognitive Decline in Adults Aged 65 and Older in Cumbaya, Quito, Ecuador: Prevalence and Risk Factors | Studies did not meet dementia criteria |
| Eldemire, D. | 1996 | Level of mental impairment in the Jamaican elderly and the issues of screening levels, caregiving, support systems, carepersons, and female burden | Studies did not meet dementia criteria |
| Ferreira-Filho, S.F., et al. | 2021 | Prevalence of dementia and cognitive impairment with no dementia in a primary care setting in southern Brazil | Studies did not meet dementia criteria |
| Feter, N., et al. | 2021 | Who are the people with Alzheimer's disease in Brazil? Findings from the Brazilian Longitudinal Study of Aging | Studies did not meet dementia criteria |
| Franca, V.F., et al. | 2018 | Diet, Epidemiological Factors and Cognitive Impairment: A Cross-Sectional Study in the Elderly Population | Studies did not meet dementia criteria |
| Gondim, A. S., et al. | 2017 | Prevalence of functional cognitive impairment and associated factors in Brazilian community-dwelling older adults | Studies did not meet dementia criteria |
| Koyanagi, A., et al. | 2018 | Chronic Physical Conditions, Multimorbidity, and Mild Cognitive Impairment in Low- and Middle-Income Countries | Studies did not meet dementia criteria |
| Laks, J., et al. | 2005 | Prevalence of cognitive and functional impairment in community-dwelling elderly - Importance of evaluating activities of daily living | Studies did not meet dementia criteria |
| Laks, J., et al. | 2003 | Mini-mental state examination in community-dwelling elderly: preliminary data from Santo Antonio de Padua, Rio de Janeiro, Brazil | Studies did not meet dementia criteria |
| Lentsck, M. H., et al. | 2015 | Prevalence of depressive symptoms and signs of dementia in the elderly in the community | Studies did not meet dementia criteria |
| Lopes, M.A., et al. | 2007 | Prevalence of cognitive and functional impairment in a community sample in Ribeirao Preto, Brazil | Studies did not meet dementia criteria |
| Lopes, M.A., et al. | 2016 | Cognitive and functional impairment in an older community population from Brazil: The intriguing association with frequent pain | Studies did not meet dementia criteria |
| Maci­as Ortega, Mónica Marcela; Paguada Canales, Elizabeth; Maradiaga, Edna; Sierra, Manuel; Hesse, Heike; Navarro, Ellen; QuiÃ±ónez, MarÃ­a J; Pineda, Angel; Andonie, Donis | 2012 | Prevalence of Dementia and Associated Factors in Older Adults, Village | Studies did not meet dementia criteria |
| Martínez-Sanguinetti, M.A., et al. | 2019 | Factors associated with cognitive impairment in older adults in Chile [Factores asociados al deterioro cognitivo en personas mayores en Chile] | Studies did not meet dementia criteria |
| Mejía Arango, S., et al. | 2020 | Cognitive Decline Among the Elderly: A Comparative Analysis of Mexicans in Mexico and in the United States | Studies did not meet dementia criteria |
| Miu, J. et al. | 2016 | Factors associated with cognitive function in older adults in Mexico | Studies did not meet dementia criteria |
| Moreno, X. et al. | 2019 | Life expectancy with and without cognitive impairment among Chilean older adults: results of the National Survey of Health (2003, 2009 and 2016) | Studies did not meet dementia criteria |
| Perales, J. et al. | 2018 | Cardiovascular health and cognitive function among Mexican older adults: cross-sectional results from the WHO Study on Global Ageing and Adult Health | Studies did not meet dementia criteria |
| Peres, M.A., et al. | 2015 | Tooth loss is associated with severe cognitive impairment among older people: findings from a population-based study in Brazil | Studies did not meet dementia criteria |
| Saenz, J.L., et al. | 2018 | Cognition and Context: Rural-Urban Differences in Cognitive Aging Among Older Mexican Adults | Studies did not meet dementia criteria |
| Manrique-Espinoza, B., Salinas-Rodríguez, A., Moreno-Tamayo, K.M., Acosta-Castillo, I., Sosa-Ortiz, A.L., Gutiérrez-Robledo, L.M., Téllez-Rojo, M.M. | 2003 | Health conditions and functional status of older adults in Mexico | Studies did not meet dementia criteria |
| Gomez, C., et al. | 2019 | Association between the Use of Proton Pump Inhibitors and Cognitive Impairment in Older Adults | Studies did not meet dementia criteria/not from LAC |
| Aguila, E, & Casanova, M | 2020 | Short-Term Impact of Income on Cognitive Function: Evidence From a Sample of Mexican Older Adults | Studies were not population or community-based |
| Benedet, A.L. et al. | 2012 | Amerindian genetic ancestry protects against Alzheimer's disease | Studies were not population or community-based |
| Hanauer, M., et al. | 2021 | Association between the functional classification of heart failure with preserved fraction ejection and cognitive impairment | Studies were not population or community-based |
| Hayashida, D.Y., et al. | 2021 | Association between baseline Mini-Mental State Examination score and dementia incidence in a cohort of oldest old | Studies were not population or community-based |
| Acosta, J.N., et al. | 2020 | Diagnosis of Rapidly Progressive Dementia in a Referral Center in Argentina | Studies were not population or community-based |
| Aguilar-Navarro, S. G. et al. | 2019 | ASSOCIATION OF VITAMIN D WITH MILD COGNITIVE IMPAIRMENT AND ALZHEIMER'S DEMENTIA IN OLDER MEXICAN ADULTS | Studies were not population or community-based |
| Aguilar-Navarro, S.G. et al. | 2019 | Spanish validation and cultural adaptation of the Five-Word Test for the detection of the neurocognitive disorder in older adults | Studies were not population or community-based |
| Aguirre-Acevedo, D.C., et al. | 2016 | Diagnostic accuracy of CERAD total score in a Colombian cohort with mild cognitive impairment and Alzheimer's disease affected by E280A mutation on presenilin-1 gene | Studies were not population or community-based |
| Alanís-Niño, G., et al. | 2008 | Prevalence of dementia in geriatric patients | Studies were not population or community-based |
| Almeida, O.P. | 1998 | The mini-mental state examination and the diagnosis of dementia in Brazil | Studies were not population or community-based |
| Almeida, Osvaldo, P. | 1998 | Mini mental state examination and the diagnosis of dementia in Brazil | Studies were not population or community-based |
| Aprahamian, I et al. | 2011 | Can the CAMCOG be a good cognitive test for patients with Alzheimer's disease with low levels of education? | Studies were not population or community-based |
| Araujo, N. B., et al. | 2018 | Diagnosing dementia in lower educated older persons: validation of a Brazilian Portuguese version of the Rowland Universal Dementia Assessment Scale (RUDAS) | Studies were not population or community-based |
| Arauz, A., et al. | 2005 | Cognitive impairment and mortality in older healthy Mexican subjects: a population-based 10-year follow-up study | Studies were not population or community-based |
| Ariza-Araujo, Y., et al. | 2017 | Dementia prevalence by stage of disease based on administrative data: Colombia (2011-2013) | Studies were not population or community-based |
| Basalo, M.M.G., et al. | 2017 | ALBA Screening Instrument (ASI): A brief screening tool for Lewy Body Dementia | Studies were not population or community-based |
| Bello-Chavolla, O.Y., et al. | 2017 | GERIATRIC SYNDROMES AND NOT CARDIOVASCULAR RISK FACTORS ARE ASSOCIATED WITH COGNITIVE IMPAIRMENT AMONG MEXICAN COMMUNITY-DWELLING ELDERLY WITH TYPE 2 DIABETES | Studies were not population or community-based |
| Bertolucci, P. H. F., et al. | 1998 | Performance of Brazilian population in neuropsychological battery of Consortium to Establish a Registry for Alzheimer's disease | Studies were not population or community-based |
| Bezerra, A.B., et al. | 2012 | School attainment in childhood is an independent risk factor of dementia in late life: results from a Brazilian sample | Studies were not population or community-based |
| Caixeta, L.F. | 2004 | Prevalence of reversible dementia in the center region of Brazil | Studies were not population or community-based |
| Capuano, A.W., et al. | 2021 | Neuroticism, negative life events, and dementia in older White and Black Brazilians | Studies were not population or community-based |
| Carvajal-Castrillón, J., et al. | 2015 | Prevalence of adult neuropsychological syndromes in a neuropsychology unit in Medellin, Colombia | Studies were not population or community-based |
| Carvalho, A.D. & Coutinho, E.D.F. | 2002 | Dementia as risk factor for severe bone fractures among the elderly | Studies were not population or community-based |
| Cecília de Melo, S., et al. | 2020 | Dementias in Brazil: Increasing burden in the 2000–2016 period. Estimates from the Global Burden of Disease Study 2016 [Demências no Brasil: Aumento da carga no período de 2000 a 2016. Estimativas do Estudo Carga Global de Doenças 2016] | Studies were not population or community-based |
| Chaves, M.L., et al. | 2009 | Incidence of Mild Cognitive Impairment and Alzheimer Disease in Southern Brazil | Studies were not population or community-based |
| Chiófalo, N., et al. | 1992 | Epidemiological study of neurological diseases in Metropolitan Santiago, Chile | Studies were not population or community-based |
| Ciciliati, A.M.M. et al. | 2021 | Severe Dementia Predicts Weight Loss by the Time of Death | Studies were not population or community-based |
| Cintra, M.T. et al. | 2016 | Advanced dementia in a sample of Brazilian elderly: Sociodemographic and morbidity analysis | Studies were not population or community-based |
| Cunha, U.G. | 1990 | An investigation of dementia among elderly outpatients | Studies were not population or community-based |
| Díaz-Cabezas, R., et al | 2006 | Neuroepidemiology profile center zone of department of Caldas (Colombia), years 2004-2005 [Perfil neuroepidemiológico en la zona centro del departamento de Caldas (Colombia), años 2004-2005] | Studies were not population or community-based |
| Duarte, P.O., et al. | 2017 | Cardiovascular risk factors and inflammatory activity among centenarians with and without dementia | Studies were not population or community-based |
| Fagundes, D.F., et al. | 2021 | Dementia among older adults living in long-term care facilities: An epidemiological study Demência em idosos residentes em instituições de longa permanência: Um estudo epidemiológico] | Studies were not population or community-based |
| Feijo, M.E.P.H., et al. | 2021 | Self-Reported Hearing Loss and Associated Factors in Older Adults at a Memory Clinic | Studies were not population or community-based |
| Fonte Sevillano, T., et al. | 2021 | Prevalence and risk factors for dementia in people 80 years or older | Studies were not population or community-based |
| Franco, J. G., et al. | 2005 | Prevalencia de trastornos psiquiátricos en pacientes medicoquirúrgicos hospitalizados en la Clínica Universitaria Bolivariana de Medellín, Colombia | Studies were not population or community-based |
| Freitas, S. et al. | 2010 | Adaptation studies of the montreal cognitive assessment (MoCA) to the portuguese population | Studies were not population or community-based |
| Garcia, A.N., et al. | 2008 | APOE-epsilon4 polymorphism and cognitive deficit among the elderly population of Fernando de Noronha | Studies were not population or community-based |
| Grinberg, L.T., et al. | 2013 | Prevalence of dementia subtypes in a developing country: a clinicopathological study | Studies were not population or community-based |
| Iturra-Mena, A.M. | 2007 | Adaptation and preliminary validation of a screening test for dementia in Chile: The eurotest [Adaptación y validación preliminar de un test para el screening de demencia en Chile: El Eurotest] | Studies were not population or community-based |
| Jacinto, A. F. | 2008 | Cognitive impairment in elderly followed by general practitioners | Studies were not population or community-based |
| Kimura, N.R.S., et al. | 2021 | Young- and Late-Onset Dementia: A Comparative Study of Quality of Life, Burden, and Depressive Symptoms in Caregivers | Studies were not population or community-based |
| Larraya, F.P., et al. | 2004 | Prevalence of dementia of the Alzheimre type, vascular dementia and other DSM-IV and ICD-10 dementias in the Republica Argentina [Prevalencia de las demencias del tipo Alzheimer, demencias vasculares y otras demencias del DSM-IV y del ICD-10 en la República Argentina] | Studies were not population or community-based |
| Leon, T., et al. | 2021 | Evaluating a Memory Clinic Using the RE-AIM Model. The Experience of the Memory and Neuropsychiatry Clinic in Hospital Del Salvador, Chile | Studies were not population or community-based |
| Lima, D. A., & Lourenco, R. A. | 2010 | Cross-cultural adaptation of section A of the Cambridge Examination for Mental Disorders of the Elderly: revised version (CAMDEX-R) for dementia diagnosis | Studies were not population or community-based |
| Machado, J. C., et al. | 2011 | Cognitive decline of aged and its association with epidemiological factors in the city of Viçosa, Minas Gerais | Studies were not population or community-based |
| Martinez, D.L., et al. | 2021 | Prevalence of behavioral and mental disorders in Manizales, Colombia | Studies were not population or community-based |
| Meguro, K., et al. | 2001 | Elderly Japanese emigrants to Brazil before World War II: II. Prevalence of senile dementia | Studies were not population or community-based |
| Meguro, K., et al. | 2011 | Incidence of dementia and cause of death in elderly Japanese emigrants to Brazil before World War II | Studies were not population or community-based |
| Molina, O. et al. | 2000 | Causes of dementia in Maracaibo, Venezuela: A reappraisal | Studies were not population or community-based |
| Moreira, I.F.H., et al. | 2009 | Cambridge Cognitive Examination: performance of healthy elderly Brazilians with low education levels/ Cambridge Cognitive Examination: desempenho de idosos brasileiros saudáveis com baixa escolaridade | Studies were not population or community-based |
| Moreno, D.J., et al. | 2017 | Association of GWAS Top Genes With Late-Onset Alzheimer's Disease in Colombian Population | Studies were not population or community-based |
| Moreno-Zambrano, D; Penaherrera-Oviedo, C; Santibanez-Vasquez, R | 2016 | Prevalence of neurological diseases in a primary care center in the Suscal Canton of Ecuador | Studies were not population or community-based |
| MuÃ±oz-Neira, C., et al. | 2012 | Psychometric properties and diagnostic usefulness of the Addenbrooke's Cognitive Examination-revised in a Chilean elderly sample | Studies were not population or community-based |
| Neri, V.C., et al. | 2021 | Prevalence study of dementia with Lewy bodies in a Parkinson's disease reference center: Clinical and laboratory analysis of a Rio de Janeiro/Brazil cohort | Studies were not population or community-based |
| Nunes, P.V., et al. | 2019 | Neuropsychiatric Inventory in Community-Dwelling Older Adults with Mild Cognitive Impairment and Dementia | Studies were not population or community-based |
| Oliveira, F.F., et al. | 2012 | Epidemiology of dementia in Sao Paulo, Brazil: risk factors and age of onset of dementia due to Alzheimer's disease | Studies were not population or community-based |
| Oliveira, F.F., et al. | 2014 | Assessment of risk factors for earlier onset of sporadic Alzheimer's disease dementia | Studies were not population or community-based |
| Pérez Martinez, V. | 2004 | Prevalence of demential syndrome in the population over 60 | Studies were not population or community-based |
| Perroco, T.R. et al. | 2009 | Performance of Brazilian long and short IQCODE on the screening of dementia in elderly people with low education | Studies were not population or community-based |
| Perrote, F.M., et al. | 2017 | Association between subjective loss of memory, mild cognitive impairment and dementia [Asociación entre pérdida subjetiva de memoria, deterioro cognitivo leve y demencia] | Studies were not population or community-based |
| Piriz, A., et al. | 2018 | Cerebrovascular Disease and Neurodegeneration in Alzheimer's Disease with and without a Strong Family History: A Pilot Magnetic Resonance Imaging Study in Dominican Republic | Studies were not population or community-based |
| Pradilla Ardila, G. et al. | 2002 | Prevalence of neurological diseases in Aratoca, a rural area of eastern Colombia [Estudio neuroepidemiológico en Aratoca, una área rural del oriente colombiano] | Studies were not population or community-based |
| Pradilla, G., et al. | 2003 | National neuroepidemiological study in Colombia (EPINEURO) | Studies were not population or community-based |
| Prat de Magalhaes, G.A. et al. | 2017 | A clinical registry of rapidly progressive dementias in a center of high complexity: towards a more accurate diagnosis of autoinmune dementias [Estudio de registro de las demencias rápidamente progresivas en un centro de alta complejidad: hacia un diagnóstico adecuado de las demencias autoinmunes en nuestro país] | Studies were not population or community-based |
| Sanchez Abraham, M., et al. | 2015 | Incidence of early-onset dementia in Mar del Plata | Studies were not population or community-based |
| Sanchez, M.A., Lourenço, R.A. | 2013 | Screening for dementia: Brazilian version of the Informant Questionnaire on Cognitive Decline on the Elderly and its psychometric properties | Studies were not population or community-based |
| Schultz, R. R., et al. | 2019 | Prevalence of dementia among widowed and non-widowed patients and associated clinical and sociodemographic characteristics/ Prevalência, caracterÃ­sticas clÃ­nicas e sociodemográficas em pacientes viúvos e nÃ£o viúvos com demência | Studies were not population or community-based |
| Souza, R.K.M., et al. | 2019 | Prevalence of dementia in patients seen at a private hospital in the Southern Region of Brazil | Studies were not population or community-based |
| Tellería-Díaz et al. | 1997 | A longitudinal study of the course of Alzheimer type dementia | Studies were not population or community-based |
| Ulloa, E.H., et al. | 2021 | Prevalence and risk factors of dementia syndrome in elderly people Prevalencia y factores de riesgo del síndrome demencial en personas mayores] | Studies were not population or community-based |
| Vardarajan, B.N. et al. | 2014 | Age-Specific Incidence Rates for Dementia and Alzheimer Disease in NIA-LOAD/NCRAD and EFIGA Families National Institute on Aging Genetics Initiative for Late-Onset Alzheimer Disease/National Cell Repository for Alzheimer Disease (NIA-LOAD/NCRAD) and Estudio Familiar de Influencia Genetica en Alzheimer (EFIGA) | Studies were not population or community-based |
| Vera-Cuesta, H., et al | 2006 | Prevalence and risk factors of age-related memory disorder in a health district [Prevalencia y factores de riesgo del trastorno de la memoria asociado a la edad en un área de salud] | Studies were not population or community-based |
| Veras, R.P., & Murphy, E. | 1994 | The mental health of older people in rio de janeiro | Studies were not population or community-based |
| Vieira, R.N., et al. | 2016 | Association between DCHS2 gene and mild cognitive impairment and Alzheimer's disease in an elderly Brazilian sample | Studies were not population or community-based |
| Vigliecca N, et al. | 2007 | Adaptation and validation of a naming test for Spanish speakers: reliability and discrimination of patients with dementia and unilateral brain lessions | Studies were not population or community-based |
| Villa, A.R., et al. | 2021 | The paradoxical effect of living alone on cognitive reserve and mild cognitive impairment among women aged 60+ in Mexico city | Studies were not population or community-based |
| Yamada, T., et al. | 2002 | Prevalence of dementia in the older Japanese-Brazilian population | Studies were not population or community-based |
| Zapata-Restrepo, L., et al. | 2021 | The Psychiatric Misdiagnosis of Behavioral Variant Frontotemporal Dementia in a Colombian Sample | Studies were not population or community-based |
| Calderón-Garcidueñas, L., et al. | 2019 | Mild Cognitive Impairment and Dementia Involving Multiple Cognitive Domains in Mexican Urbanites | Studies were not population- or community-based sample in older population |
| Ashby-Mitchell, K., et al. | 2018 | The proportion of dementia attributable to common modifiable lifestyle factors in Barbados | Studies were not population or community-based/Studies did not meet dementia criteria |
| Vergara, R.C., et al. | 2022 | Population attributable fraction of modifiable risk factors for dementia in Chile | Studies were not population or community-based/Studies did not meet dementia criteria |
| Wong-Achi, X., et al. | 2017 | Cognitive impairment in rural elderly population in ecuador | Studies were not population or community-based/Studies did not meet dementia criteria |
| Fuentes, P., & Albala, C. | 2014 | An update on aging and dementia in Chile | Studies were not population or community-based/ Theoretical, protocol, editorial, or reviews |


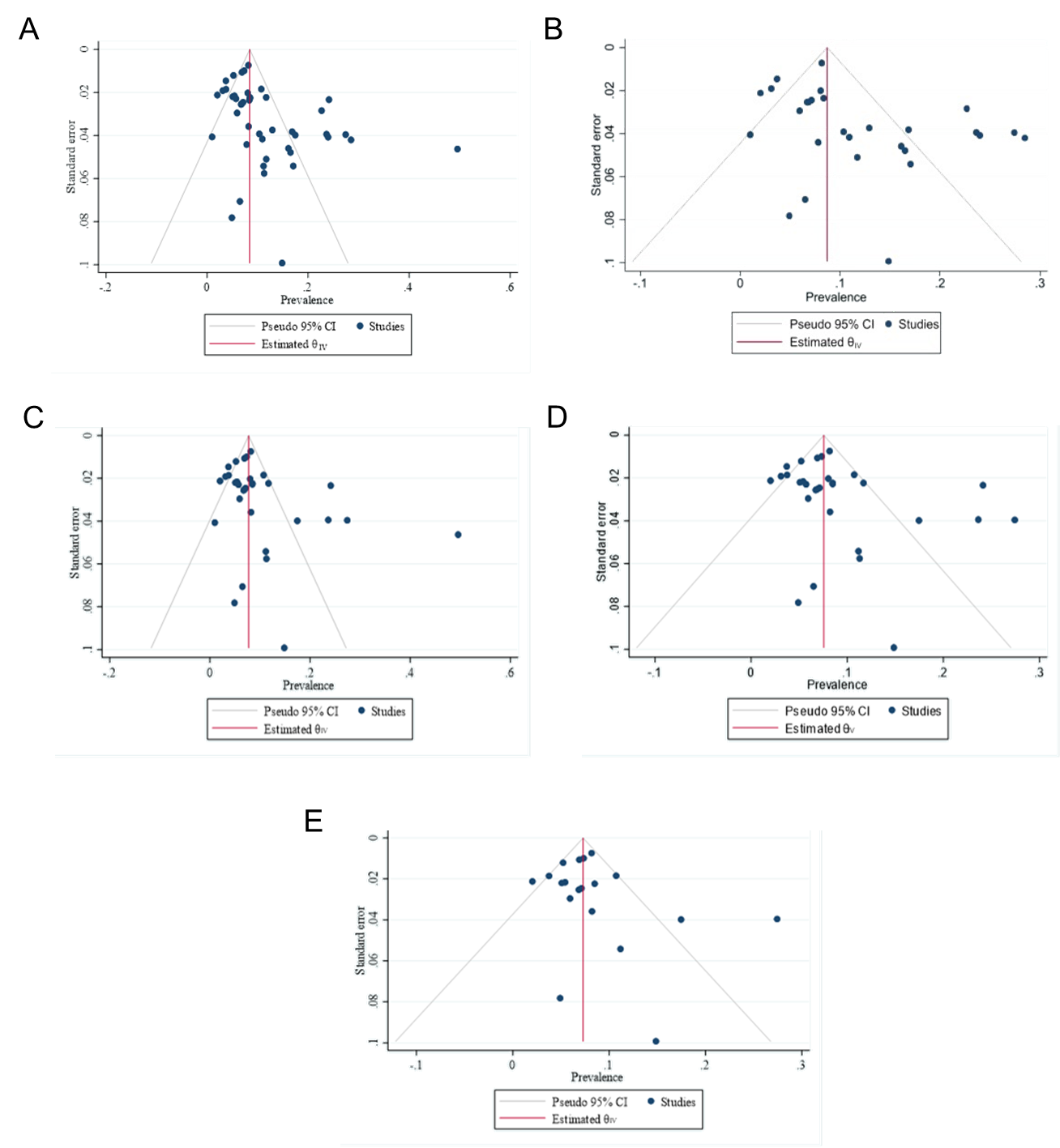


Figure S1: Funnel plots for meta-analysis of the prevalence of all-cause dementia including all selected studies (A), excluding studies with one-phase diagnosis (B), representative samples (C), after performing the leave-one-out method (D), and by countries (E).


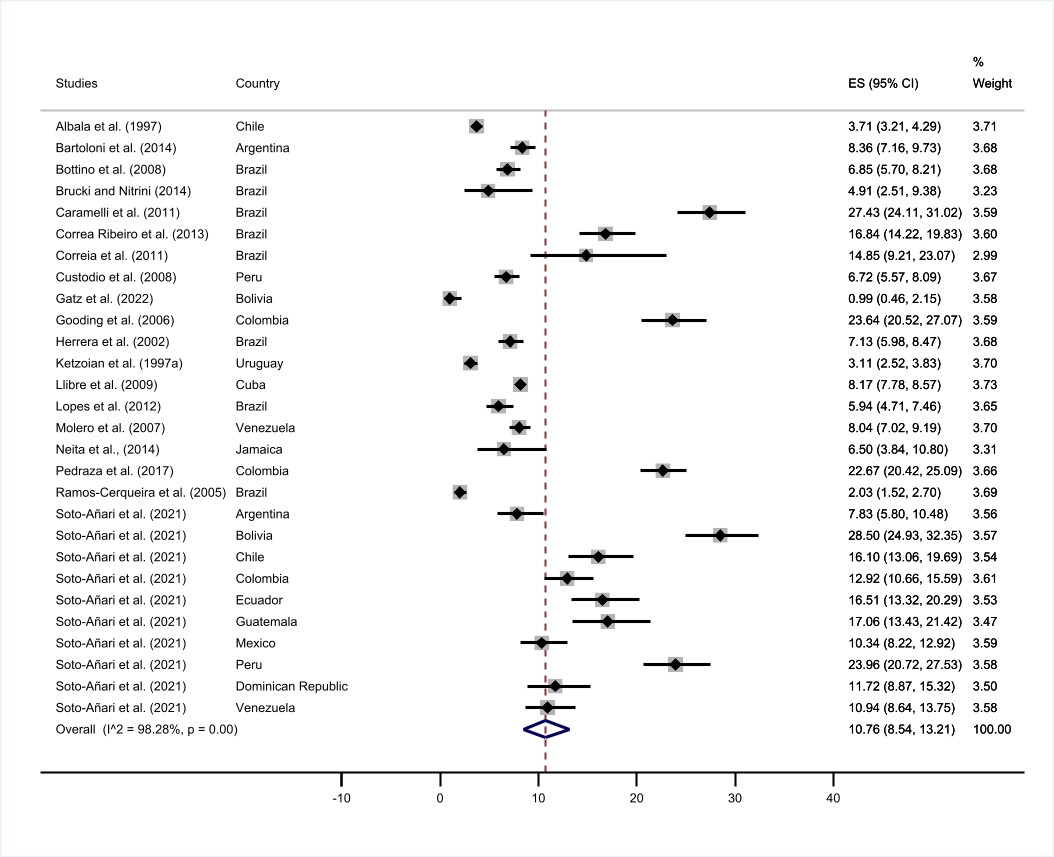


Figure S2. Meta-analysis of prevalence of all-cause dementia excluding studies with one-phase diagnosis.


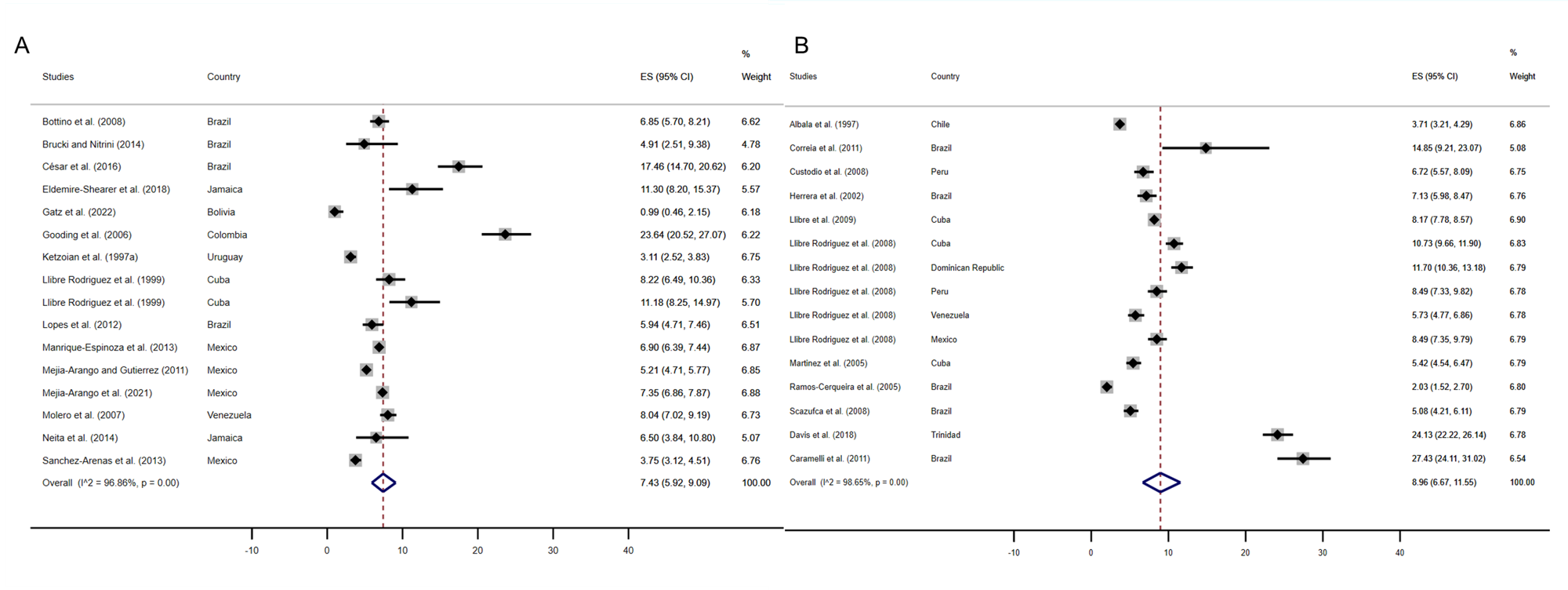


Figure S3. Meta-analysis of prevalence of all-cause dementia in studies including participants ≥ 50 years old and (A) ≥ 65 years old (B).


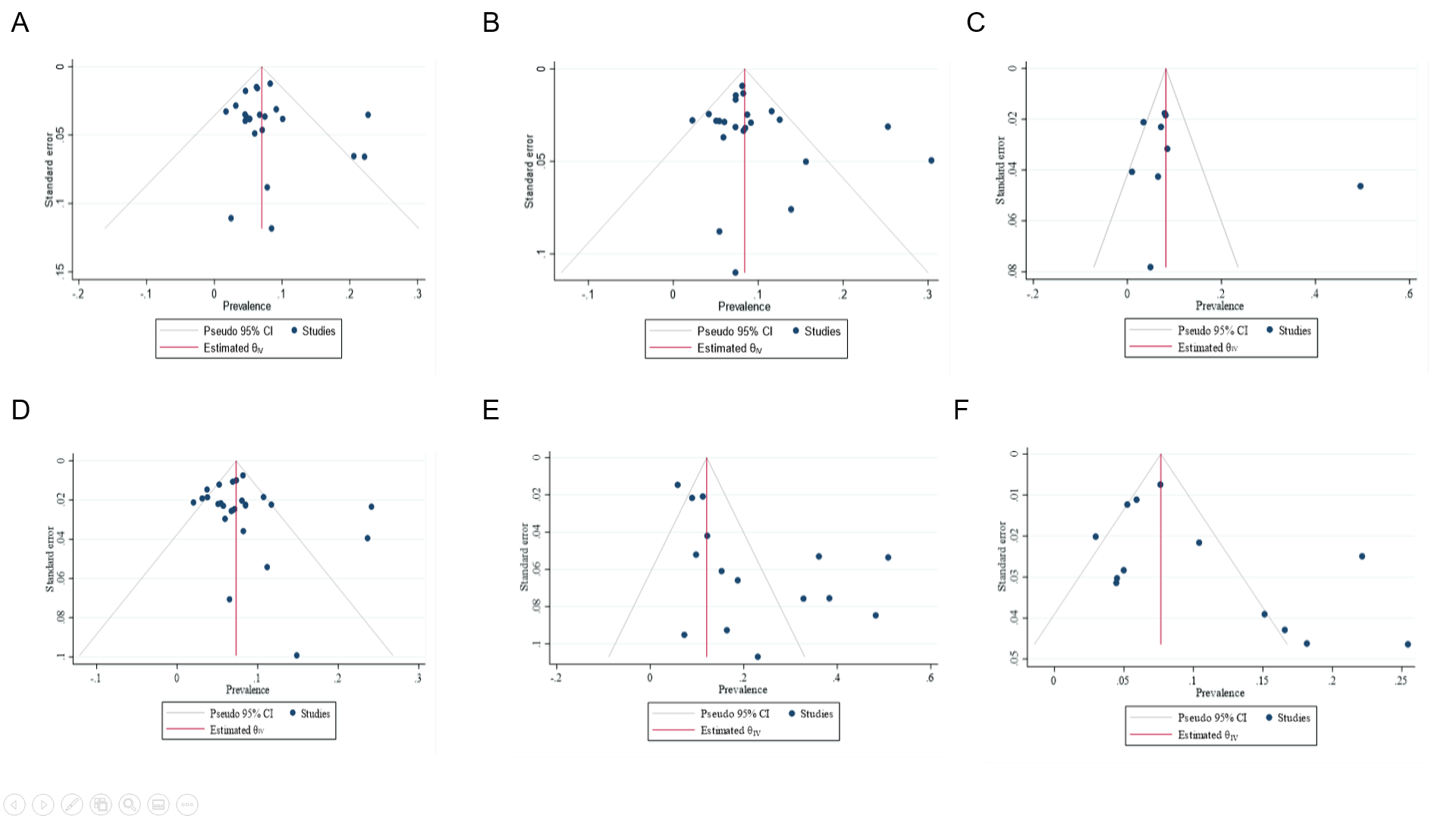


Figure S4: Funnel plots for meta-analysis of the prevalence of all-cause dementia for men to same-sex participants (A), for women to the same-sex participants (B), for participants from rural area (C), for participants from urban area (D), participants with no formal education (E), and for participants with at least one year of formal education (F).
